# Excess of body weight is associated with accelerated T-cell senescence in hospitalized COVID-19 patients

**DOI:** 10.1186/s12979-024-00423-6

**Published:** 2024-03-08

**Authors:** Mailton Prestes Madruga, Lucas Kich Grun, Letícya Simone Melo Dos Santos, Frederico Orlando Friedrich, Douglas Bitencourt Antunes, Marcella Elesbão Fogaça Rocha, Pedro Luis Silva, Gilson P. Dorneles, Paula Coelho Teixeira, Tiago Franco Oliveira, Pedro R.T. Romão, Lucas Santos, José Claudio Fonseca Moreira, Vinicius Schenk Michaelsen, Marcelo Cypel, Marcos Otávio Brum Antunes, Marcus Herbert Jones, Florencia María Barbé-Tuana, Moisés Evandro Bauer

**Affiliations:** 1 Laboratory of Immunobiology, School of Health and Life Sciences, Pontifical Catholic University of Rio Grande do Sul (PUCRS), Av. Ipiranga, 6681, building 12 (4th floor), Porto Alegre, 90619-900 RS Brazil; 2 School of Medicine, Pontifical Catholic University of Rio Grande do Sul (PUCRS), Porto Alegre, RS Brazil; 3 Laboratory of Cellular and Molecular Immunology, Federal University of Health Sciences of Porto Alegre (UFCSPA), Porto Alegre, RS Brazil; 4 Centro de Estudos em Estresse Oxidativo - Programa de Pós-Graduação em Biologia Celular e Molecular, Instituto de Biociências, Universidade Federal do Rio Grande do Sul (IB-UFRGS), Porto Alegre, RS Brazil; 5 Latner Thoracic Research Laboratories, Toronto General Hospital Research Institute, University Health Network, Toronto, Canada; 6 Toronto General Hospital Research Institute, Department of Surgery, University Health Network, University of Toronto, Toronto, Canada

**Keywords:** Ageing, SARS-CoV-2, Inflammation, Obesity, Microbial translocation, T cells

## Abstract

**Background:**

Several risk factors have been involved in the poor clinical progression of coronavirus disease-19 (COVID-19), including ageing, and obesity. SARS-CoV-2 may compromise lung function through cell damage and paracrine inflammation; and obesity has been associated with premature immunosenescence, microbial translocation, and dysfunctional innate immune responses leading to poor immune response against a range of viruses and bacterial infections. Here, we have comprehensively characterized the immunosenescence, microbial translocation, and immune dysregulation established in hospitalized COVID-19 patients with different degrees of body weight.

**Results:**

Hospitalised COVID-19 patients with overweight and obesity had similarly higher plasma LPS and sCD14 levels than controls (all *p* < 0.01). Patients with obesity had higher leptin levels than controls. Obesity and overweight patients had similarly higher expansions of classical monocytes and immature natural killer (NK) cells (CD56^+^CD16^−^) than controls. In contrast, reduced proportions of intermediate monocytes, mature NK cells (CD56^+^CD16^+^), and NKT were found in both groups of patients than controls. As expected, COVID-19 patients had a robust expansion of plasmablasts, contrasting to lower proportions of major T-cell subsets (CD4 + and CD8+) than controls. Concerning T-cell activation, overweight and obese patients had lower proportions of CD4^+^CD38^+^ cells than controls. Contrasting changes were reported in CD25^+^CD127^low/neg^ regulatory T cells, with increased and decreased proportions found in CD4^+^ and CD8^+^ T cells, respectively. There were similar proportions of T cells expressing checkpoint inhibitors across all groups. We also investigated distinct stages of T-cell differentiation (early, intermediate, and late-differentiated – TEMRA). The intermediate-differentiated CD4 + T cells and TEMRA cells (CD4^+^ and CD8^+^) were expanded in patients compared to controls. Senescent T cells can also express NK receptors (NKG2A/D), and patients had a robust expansion of CD8^+^CD57^+^NKG2A^+^ cells than controls. Unbiased immune profiling further confirmed the expansions of senescent T cells in COVID-19.

**Conclusions:**

These findings suggest that dysregulated immune cells, microbial translocation, and T-cell senescence may partially explain the increased vulnerability to COVID-19 in subjects with excess of body weight.

**Supplementary Information:**

The online version contains supplementary material available at 10.1186/s12979-024-00423-6.

## Background

Although most cases of coronavirus disease 19 (COVID-19) are asymptomatic or mild, severe COVID-19 is characterised by hyperinflammation, immune dysregulation, acute respiratory distress syndrome, and hypercoagulation, which may result in multiple organ failure and death [1]. Several clinical risk factors have been associated with poor disease progression, including ageing, male sex, diabetes, and excess of body weight [2]. Indeed, ageing of the immune system (immunosenescence) has been proposed to be involved with poor COVID-19 outcomes, as highlighted by increased mortality rates observed among older adults [3]. Obesity is considered a model of premature immunosenescence and it has also been associated with poor COVID-19 outcomes [2, 4, 5]. A large cohort study evaluated data from 6,910,695 adults with COVID-19 and reported that obesity is enough to cause adverse events in the course of the disease, regardless of other comorbidities [2]. Thus, obesity alone is a major risk factor for in-hospital mortality with potential negative impact factor outweighing that of cardiovascular disease and diabetes [6]. Younger obese patients (20 to 39 years), vaccinated or not, had severe COVID-19 and more adverse events, even when compared with older adults.

Ageing is associated with chronic low-grade inflammation (termed “inflammaging”) and is associated with the development of chronic conditions and death [6]. Like ageing, there is a systemic elevation of inflammatory mediators in obesity that may contribute to early immunosenescence [7, 8]. Plasma from obese subjects can induce in vitro cellular senescence in peripheral blood mononuclear cells (PBMCs) from eutrophic individuals [8]. Both ageing and obesity are associated with the expansion of late differentiated (senescent) T cells (CD28^−^ or CD28null) in the circulation, providing the stage for the development of hypertension, type II diabetes, non-alcoholic steatosis, and cancer [7, 8].

Significant advances have been made in understanding how COVID-19 affects the human physiology. The immunopathology of COVID-19 was characterised by dysregulated innate responses, as shown by the augmented secretion of pro-inflammatory mediators (ex., tumour necrosis factor (TNF)-α, interleukin (IL)-1, IL-6, and C-reactive protein, CRP), excess of immature neutrophils and activated inflammasome, and lower levels of type I and III interferons [9]. In addition, the SARS-CoV-2 induces pulmonary senescence, triggering paracrine inflammation, and leading to a sustained senescence-associated inflammatory response [10, 11]. While the respiratory system serves as the primary target of COVID-19 infection, the pathogenesis may extend to various organs and systems, including the heart, kidneys, blood vessels, liver, and intestines [9]. Emerging evidence indicates that severe COVID-19 is associated with gut barrier disruption contributing to the spread of microbial products and impacting the host’s immune response. An altered gut-lung axis during SARS-CoV-2 infection through hyperactive inflammatory responses may lead to lung hyper-permeability, gut infection, and disruption of intestinal permeability. Indeed, it has been shown recently that ICU COVID-19 patients had increased peripheral markers of bacterial translocation (i.e., lipopolysaccharide (LPS) and soluble CD14), which were associated with inflammation and mortality [12, 13]. In addition to hyperinflammation, severe COVID-19 has several features of dysregulated adaptive immune responses. Indeed, COVID-19 is associated with lymphopenia and the accumulation of exhausted circulating T and B cells. The extent of lymphopenia (of note in CD8^+^ T cells) in patients admitted to the ICU seemingly correlates with COVID-19 severity and mortality [14, 15]. After SARS-CoV-2 infection, severe disease is also characterized by activated T cells with an effector memory or exhausted phenotype (CD27^−^CD28^−^CD45RA^+^CD57^+^KLRG1^+^), primarily in CD8^+^ T cells [16, 17].

To date, there is a lack of COVID-19 studies that have analysed the impact of overweight and obesity on innate and adaptive immune mechanisms, specifically focusing on immunosenescence. T-cell markers of exhaustion, inhibition, and apoptosis have been found implicated in previous studies correlating immunosenescence in cancer or persistent viral infections: NKG2A (senescence), PD-1 (apoptosis), TIM-3, CTLA-4 and LAG-3 (exhaustion) [18, 19]. Therefore, this study aims to investigate whether accelerated immunosenescence and immune dysregulation are established in obese subjects hospitalized with COVID-19.

## Methods

### Study population

Hospitalized COVID-19 patients with confirmed SARS-CoV-2 RT PCR on nasopharyngeal specimens (*n* = 50), receiving low-flow supplemental oxygen (maximum of four litres per minute) (WHO Clinical Progression Scale score 4, hospitalized with supplemental oxygen) (WHO 2020), were recruited within 7 to 10 days of symptoms. Subjects were accessed in this study no later than 48 h after entering the emergency room. Patients were admitted to the COVID-19 Unit of Hospital São Lucas at PUCRS (Porto Alegre/RS, Brazil) between December/2020 and March/2022. Of the 50 patients, 15 receive at least one dose of COVID-19 vaccine. Written informed consent was obtained from all subjects and the study was approved by the PUCRS Institutional Review Board (#34578820.6.1001.5336). Electronic medical records with clinical and sociodemographic data were collected and stored using the REDCap software. Blood samples were also obtained from 11 age- and sex-matched SARS-CoV-2 RT-PCR negative controls.

### Blood collection and isolation of peripheral blood mononuclear cells

Blood samples were collected from all subjects from the antecubital vein in 8 mL EDTA tubes within 48 h after admission at the hospital. Plasma samples were centrifuged (450 *g*, 5 min), aliquoted, and immediately kept at -80 ºC until analysis. Peripheral blood mononuclear cells (PBMCs) were isolated by a Ficoll-Hypaque gradient (400 *g*, 30 min). Cells were counted by microscopy (×100) and viability always exceeded 98%, judged by their ability to exclude Trypan Blue (Sigma, St. Louis, MO). PBMCs were resuspended in the freezing media (90% fetal bovine serum and 10% Dimethyl Sulfoxide), kept at -80ºC for 24 h, and then cryopreserved in liquid nitrogen (-196ºC) until analysis.

### Liquid chromatography-tandem mass spectrometry determination of LPS

The concentration of LPS was determined using liquid chromatography-tandem mass spectrometry (LC-MS/MS), employing the quantification of 3-hydroxytetradecanoic acid as described by Pais de Barros and colleagues [20]. Briefly, 150 μL of plasma underwent hydrolysis with 75 μL of 150 mM NaCl and 300 μL of 8 mol HCl for 4 h at 90 ◦C, followed by extraction with 4 mL of hexane. Subsequently, the samples were dried under a nitrogen stream and reconstituted in 50 μL of methanol immediately before injection into the analytical system. The analysis was conducted using a Nexera UFLC system coupled to an LCMS-8045 triple quadrupole mass spectrometer (Shimadzu, Kyoto, Japan). Electrospray parameters were configured in the negative ion mode with the following settings: capillary voltage, 3000 V; desolvation line temperature, 250 ◦C; heating block temperature, 500 ºC; drying gas, 18 L/min; and nebulizing gas, 2 L/min. Collision-induced dissociation was achieved with 230 kPa argon pressure. Multiple reaction monitoring (MRM) was employed with the fragmentation transition of m/z 243.1 → m/z 59.0. The chromatographic separation was conducted with an Acquity UPLC® C18 column (2.1 × 50 mm, 1.7 μm particle size) (Waters Corporation, Ireland). The analyses were performed in gradient elution mode with a flow rate of 0.4 mL/min and the gradient mobile phase system consisted of water (solvent A) and acetonitrile (solvent B) both fortified with 0.2% acetic acid as follows: 0–1.5 min, 75–100% of B; 1.5–2.0 min, 100% of B; 2.0–2.1 min, 100–75% of B; 2.1–5.5 min, 75% B. The column oven was maintained at 50 ◦C. Data processing was conducted using LabSolutions software (Shimadzu, Kyoto, Japan), and calibration curves were constructed within the range of 0.2–1000 ng/mL.

### Plasma adipokines and sCD14

MILLIPLEX® bead-based multiplex immunoassays were performed to detect and quantify multiple protein targets. The MAGPIX™ platform was used to detect plasma Adiponectin (MILLIPLEX® MAP Human Adipokine Magnetic Bead Panel 1, Millipore, São Pa), and Active Ghrelin and Leptin (MILLIPLEX® MAP Human Metabolic Hormone Magnetic Bead Panel), accordingly to the manufacturer’s instructions for each kit. All kits were purchased from Merck-Millipore (São Paulo, Brazil). The detection limits for these assays were: adiponectin (11–400,000 pg/mL), leptin (41–100,000 pg/mL) and ghrelin (14–10,000 pg/mL). The soluble form of CD14 (sCD14) was analysed using a Human CD14 ELISA Kit from Invitrogen (USA), with detection limits ranging from 8.23 to 8,000 ng/mL.

### Immunophenotyping

Cryopreserved PBMCs were thawed rapidly in a 37 °C water bath and washed in media (RPMI-1640 supplemented with 10% FBS and 1% penicillin-streptomycin) by centrifuging at 400 *g* for 5 min. This study included a comprehensive multicolour immunophenotyping analysis of different panels aimed at analysing both innate cells (monocytes, NK cells) and adaptive immune cells (B, T, NKT), including their activation state, differentiation stages, regulatory profiles, senescence, and exhaustion profiles. On the day of analysis, cells were washed with cytometry buffer (PBS containing 1% FBS and 0.01% sodium azide) and stained for 30 min with the combination of monoclonal antibodies (all from Becton Dickinson (BD) Biosciences, San Jose, USA). For intracellular staining (granzyme B, GZB), samples were fixed in Fixation Buffer (BD CytoFix, BD Biosciences, San Jose, USA) for 20 min at 4 °C, followed by a wash with BD FACS buffer (400 *g*, 5 min, 4 °C). The cells were then permeabilized (BD Perm Buffer III) for 30 min at 4 °C. The permeabilized samples were then stained with a specific anti-granzyme-PE antibody (30 min, 4 °C).

Different panels were used to evaluate specific subpopulations of monocytes, and lymphocytes: Monocyte Subsets and NK (panel #1), B-cell subsets (panel #2), T-cell Differentiation Stages (panel #3), T-cell Activation/Regulatory (panels #4 and #5), T-cell Senescence (panels #6 and #7), and Immune checkpoint inhibitors/exhaustion (panel #8). See Table S1 for antibody panel information.

### Flow cytometry analysis

After staining, cells were washed, resuspended, and analysed in a BD FACSCanto™ II (3-Laser Violet/Blue/Red) in a 4-2-2 colour configuration (BD Biosciences). A minimum of 30,000 events were identified by size and granularity. Voltages were optimised and maintained for all samples. Compensation was performed each day using single-stained tubes and positive and negative compensation beads (BD Biosciences, São Paulo, Brazil) and parameters were assessed automatically (BD FACS DIVA™, BD, USA). Data analysis was performed with FlowJo 10.2 software (BD Biosciences, USA), using the gating strategy shown in Figure S1 in Supplementary Material. The relative expression of cell-surface markers was assessed by the analysis of the median fluorescence intensity (MFI), and Figure S2 shows the fluorescence of negative/positive controls for all markers.

Human monocytes were gated accordingly to FSC-A and SSC-A parameters and sub-grouped based on the relative expression of CD14 and CD16 into classical (CD14^bright^CD16^−^), intermediate (CD14^+^CD16^+^), and nonclassical (CD14^+^CD16^bright^) phenotypes [21]. NK cells (CD3-CD56+) were sub-grouped into immature NK cells (CD56^+^CD16^−^) and mature NK cells (CD56^+^CD16^+^) [22]. B cells (CD3^−^CD19^+^) were further defined into innate-like B cells (CD3CD19^+^CD21^+^CD38^+^), memory B cells (CD3CD19 + CD27^+^CD38^−^), regulatory-like cells (CD3^−^CD19^+^CD38^bright^), and plasmablasts (CD3CD19^+^CD27^+^CD38^+^) [23].

CD3^+^ T cells were identified as CD4^+^, CD8^+^ and NKT (CD3^+^CD56^+^) cells, and further refined into early (CD25^+^, CD25^+^CD69^+^, CD38^+^, CD69^+^) or late-activated (HLA-DR^+^CD38^+^) cells. Cytotoxic T cells were defined as CD3^+^CD8^+^CD38^+^GZB^+^. Regulatory T cells were defined as CD3^+^CD4^+^CD25^+^CD127^low/neg^ cells [24]. The following checkpoint inhibitors, involved with T-cell maturation and exhaustion, were defined in either CD4^+^ or CD8^+^ subsets: PD-1, TIM-3, LAG-3, and CTLA-4. Different stages of T-cell differentiation were determined based on the cell-surface expression of the costimulatory molecules CD27, CD28, CD57 and CD45RA [25]. This strategy discriminated early differentiated T cells (CD27^+^CD28^+^CD57^−^CD45RA^−^), naïve T cells (CD27^+^CD28^+^CD57^−^CD45RA^+^), intermediate differentiated T cells (CD27^−^CD28^+^CD57^+^CD45RA^−^), and late-differentiated or senescent T cells (CD27^−^CD28^−^ CD57^+^CD45RA^+^_,_ TEMRA). T-cell senescence can be also described by the expression of NK receptors (NKG2A/D) [26, 27]. Therefore, we identified subsets defined as NKG2A^+^ or NKG2D^+^ T cells evaluated in either CD4^+^ or CD8^+^ subsets, with or without additional markers involved with cell maturity or senescence (CD57, PD-1).

### Unsupervised analysis of two flow cytometry panels

In addition to performing traditional analysis by manual gating on cytometry data, we conducted a high-dimensional flow cytometric analysis on two relevant panels: (1) T-cell differentiation stages and (2) senescence. FlowJo (FlowJo, LLC, USA) was used for data analysis and visualization. Data from the three groups were concatenated accordingly to their two panels. Data from each file provided 1,000 events and debris were excluded. This analysis yielded a concatenated file with a total of 61,000 events. With the concatenated file, we employed the Uniform Manifold Approximation and Projection (UMAP) method to reduce dimensionality and generate a comprehensive two-dimensional (2D) graph for visualization. UMAP was chosen due to its essential ability to preserve the global structure and performance, without limitations in the incorporation of dimensions [28, 29]. Heat maps finally report statistical analysis. The cells were then grouped based on their phenotypes homogeneously using Phenograph (FlowJo S/W plugin) with a k value of 30 [30]. Other values and factors such as k-nearest neighbors (KNN) were maintained as in previous studies [31]. We incorporate information such as FSC-A, SCC-A, and all antibodies used in the panels into UMAP, and subsequently into the Phenograph algorithm. The analyses were performed separately according to each panel.

The data presented in the Stages of T-Cell Differentiation panel employed a strategy to identify major cell types and their T-cell subsets. These subsets were subsequently determined based on the expression of the molecules CD3, CD4, CD8, CD27, CD28, CD57 and CD45RA. Thirty-two meta clusters were found, encompassing 15 clusters of T cells classified into early, intermediate, and senescent. The data presented in Senescence Panel 1 employed a strategy to identify major cell types and their T and NK cell subsets. These subsets were subsequently determined based on the expression of CD3, CD56, CD8, as well as the assessment of increased expression of CD57, NKG2A, and PD-1. At baseline, 31 meta clusters were generated, comprising 5 CD8 clusters, 2 CD8/CD56 clusters, 3 NK clusters, 1 NKT cluster, 6 CD3-only clusters, and 14 CD3-negative clusters. Group comparisons within each cluster were performed by One-Way (ANOVA) or Kruskal-Wallis.

### Statistical analysis

All variables were checked for normality of distribution using the Kolmogorov-Smirnov. For categorical variables, differences between groups were compared using chi squared (X^2^) and Fisher’s exact test. For continuous variables, differences between groups were performed by ANOVAs or Kruskal-Wallis, when indicated. Pearson and Spearman’s tests were used for correlation analysis. Statistical analysis and graphical presentations were performed using Jamovi 2.3.28 (open statistical software) and GraphPad Software 9.5 (GraphPad Software Inc, La Jolla, CA). The significance level was set at α = 0.05 (two-tailed).

## Results

### Characteristics of the study populations

Demographic and clinical characteristics are shown in Table 1. Subjects were grouped into non-infected controls (*n* = 11), overweight COVID-19 (*n* = 22) and obese COVID-19 (*n* = 28). Groups did not differ concerning sociodemographic variables (age, sex, and ethnicity). The mean length of hospital admission was 9.4 (3–28) days for the overweight group and 11 (3–46) for obese patients. The obese group had six of the individuals admitted to the ICU compared with three of the overweight group (*p* = 0.71). Mortality did not differ between overweight (*n* = 3 deaths) and obese (*n* = 2 deaths) groups, *p* = 0.64. As expected, the subjects who died (66.8 yrs) were older than those who survived (53.3 yrs), *p* < 0.05. COVID-19-related symptoms were similar between the two groups (all *p* = NS): fever (70%), dyspnoea (92%), cough (90%), sore throat (38%), flu-like syndrome (100%), fatigue (88%), nausea or vomiting (34%), diarrhoea (60%), headache (70%), and coryza (34%). Also, the proportions of subjects with medical conditions did not vary between patients (all *p* = NS): hypertension (44%), diabetes mellitus (12%), Parkinson (2%), major depression (6%). Patients were under a similar pharmacological treatment (all *p* = NS): azithromycin (52%), glucocorticoids (47%), hydroxychloroquine or chloroquine (12%), anti-angiotensin II (18%), and anti-ACE (16%).

**Table 1:** Clinical and sociodemographic characteristics. Group comparisons were performed by Mann–Whitney U-test or Kruskal-Wallis, when indicated

		COVID-19		
	Controls	All	Overweight	Obese
	(*n* = 11)	(*n* = 50)	(*n* = 22)	(*n* = 28)
Age (years)	53.7 (32–90)	54.6 (31–85)	54.6 (31–84)	54.7 (31–85)
Sex (female)	4	18	5	13
Body mass (kg)	76.6 (48–99)	90 (59–135) ^A^	82 (59–100)	96.4 (74–135) ^A,B^
BMI (all)	26.6 (21.9–31.4)	30.9 (24.2–46.1) ^A^	27.3 (25–29.8)	33.8 (30.0–46.1) ^A,B^
Females	25.3 (21.9–29.1)	31.7 (25.0–38.0)	26.8 (25.2–29.1)	33.6 (30.0–38.0)
Males	27.3 (22.5–31.1)	30.5 (24.2–46.1)	27.4 (25–29.8)	34.0 (30.1–46.1)
Ethnicity (%)				
White	54.5	78	95.4	21.4
Brown	21.2	14		17.8
Black	18.1	8	4.5	60.7
Length of in-hospital stay (days)	--	10.3 (3–46)	9.4 (3–28)	11 (3–46)
Clinical score (WHO)	--	2.9 (1–8)	2.9 (1–8)	2.9 (1–7)
Ghrelin (mg/dL)	0.03 (0–0.10)	0.018 (0–0.14)	0.0069 (0–0.03)	0.028 (0–0.14)
Leptin (mg/dL)	2053 (520.1–4204)	3878 (1005–8973)	3473 (1011–8662)	4163 (1005–9973) ^A^
Adiponectin (mg/dL)	2568 (2021–2996)	2555 (2050–3153)	2605 (2050–3153)	2517 (2092–2930)

Data are presented as mean (min - max). **A** Denotes statistical difference compared to Controls (*p* < 0.05). **B** Denotes statistical difference compared to COVID-19 overweight (*p* < 0.05)

We have assessed plasma ghrelin and adipokines, previously involved with obesity as well as clinical progression and outcomes in COVID-19 (Table 1) [32]. COVID-19 patients with obesity had higher leptin levels than controls (*p* < 0.05), albeit with no changes in adiponectin and ghrelin.

### Increased markers of microbial translocation in COVID-19

**Fig. 1:**
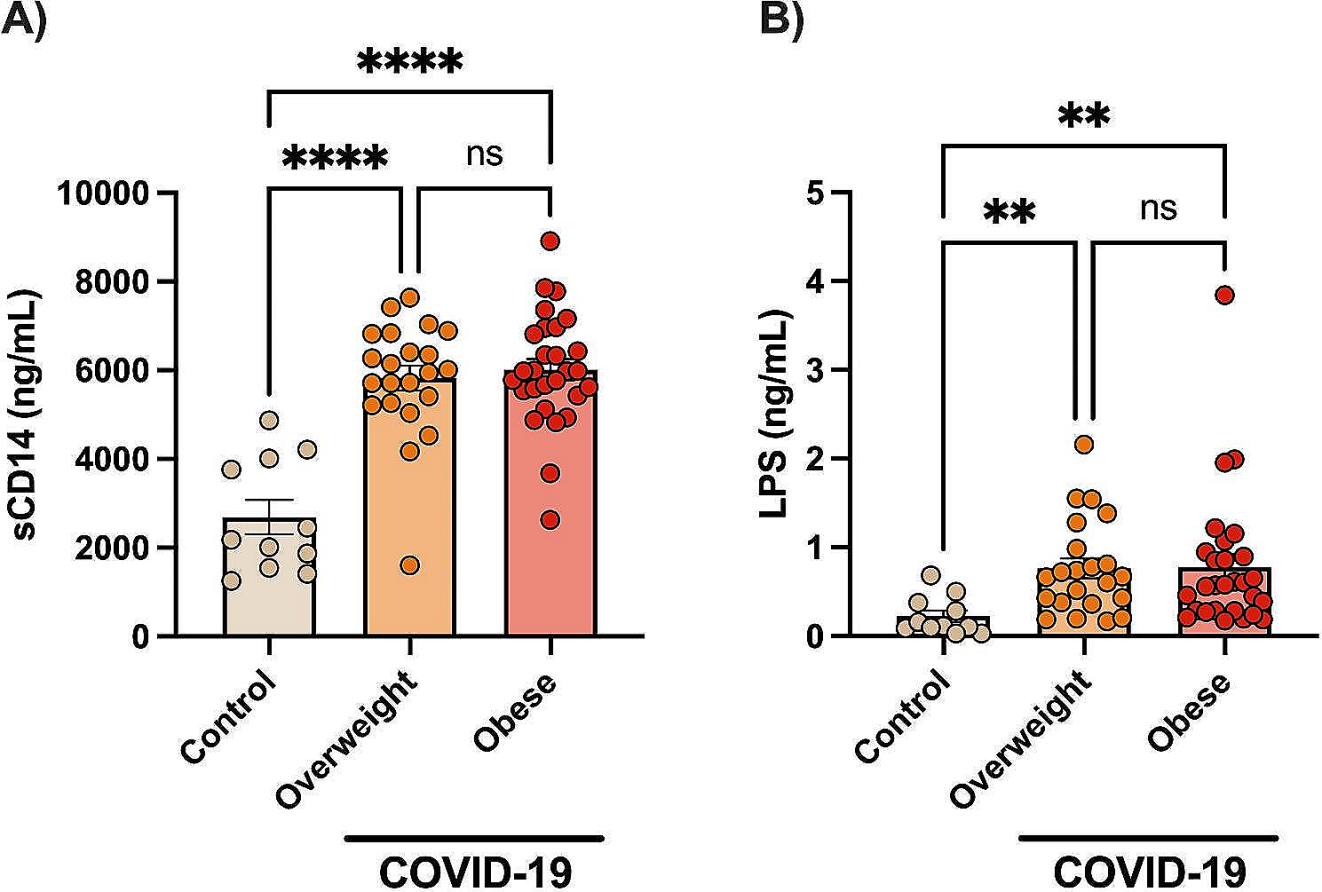
Increased circulating markers of microbial translocation in hospitalized COVID-19 patients. Data are shown as mean ± SE and scatter plots. Statistically significant differences are indicated: ** *p* < 0.001 and **** *p* < 0.0001 (Kruskal-Wallis, Dunn’s multiple comparison test)

Plasma LPS and sCD14 levels were investigated here as markers of microbial translocation. Figure 1 shows that COVID-19 patients with obesity and overweight had similarly higher LPS (*p* < 0.001) and sCD14 levels (*p* < 0.0001) than controls.

### Dysregulated innate immune cells in COVID-19

**Fig. 2:**
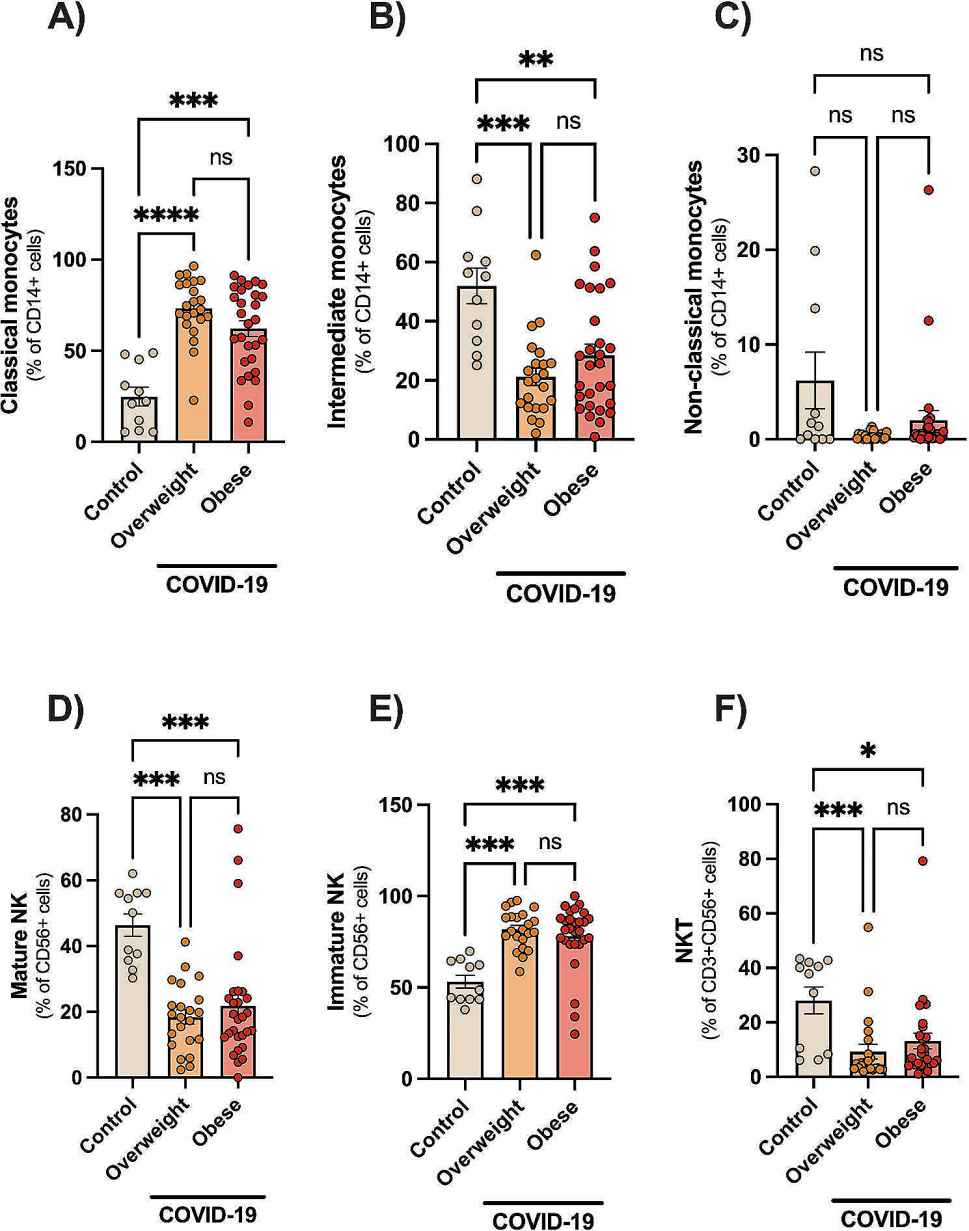
Dysregulated innate immune cells in hospitalized COVID-19 patients. Human monocytes were sub-grouped based on the relative expression of CD14 and CD16 into classical (CD14^bright^CD16^−^), intermediate (CD14^+^CD16^+^), and nonclassical (CD14^+^CD16^bright^) phenotypes. Data are shown as mean ± SE and scatter plots. Statistically significant differences are indicated: * *p* < 0.05, ** *p* < 0.001 and **** *p* < 0.0001 (Kruskal-Wallis, Dunn’s multiple comparison test)

Next, we characterized different monocyte and natural killer (NK) cell subsets by multicolour flow cytometry (Fig. 2). Human monocytes are frequently sub-grouped based on the relative expression of CD14 and CD16 into classical (CD14^bright^CD16^−^), intermediate (CD14^+^CD16^+^), and nonclassical (CD14^+^CD16^bright^) phenotypes [21]. Both, overweight and obese patients had similarly higher expansions of classical monocytes (Fig. 2A) and immature NK cells (CD56^+^CD16^−^, Fig. 2E) than controls (all *p* < 0.001). In contrast, reduced proportions of intermediate monocytes (Fig. 2B), mature NK cells (CD56^+^CD16^+^, Fig. 2D), and NKT (CD3^+^CD56^+^, Fig. 2F) were found in both groups of patients than controls (all *p* < 0.001). No changes in non-classical monocytes were observed (Fig. 2C).

### Expansion of plasmablasts in contrast to reduced CD4 + and CD8 + T cells in COVID-19

**Fig. 3:**
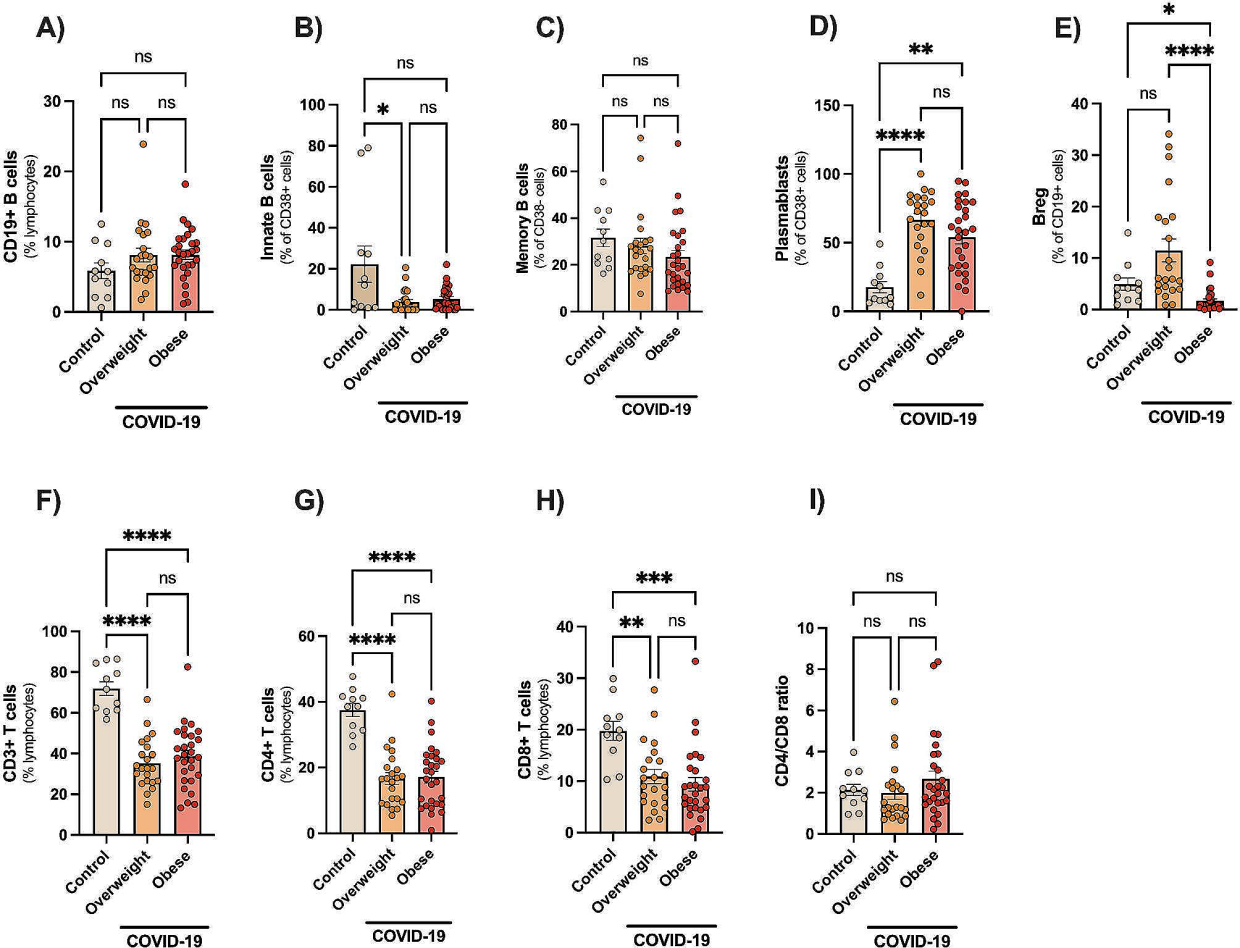
Expansion of plasmablasts in contrast to reduced CD4 + and CD8 + T cells in hospitalized COVID-19 patients. Human B cells were sub-grouped as follows: total B cells (CD3^−^CD19^+^), innate-like B cells (CD3CD19^+^CD21^+^CD38^+^), memory B cells (CD3CD19 + CD27^+^CD38^−^), regulatory-like cells (CD3^−^CD19^+^CD38^bright^), and plasmablasts (CD3CD19 + CD27^+^CD38^+^). Data are shown as mean ± SE and scatter plots. Statistically significant differences are indicated: * *p* < 0.05, ** *p* < 0.001 and **** *p* < 0.0001 (Kruskal-Wallis, Dunn’s multiple comparison test)

Figure 3 shows major B and T-cell subsets in peripheral blood. Both groups of COVID-19 patients showed a robust expansion (3-fold increase) of plasmablasts (Fig. 3D, all *p* < 0.01), contrasting to lower proportions (-2-fold decrease) of major T-cell subsets (CD4 + and CD8+) than controls (Fig. 3F and G, all *p* < 0.01). No changes in CD4/CD8 ratio were reported (Fig. 3I). Only overweight COVID-19 patients had lower proportions of innate-like B cells (CD19^+^CD21^+^CD38^+^) than controls (Fig. 3B). In addition, only patients with obesity had reduced proportions of B regulatory-like cells (CD19^+^CD38^bright^) (Fig. 3E) than overweight (*p* < 0.0001) or control (*p* < 0.05) groups.

### Dysfunctional activated and regulatory T cells in COVID-19

Table 2 shows different immune phenotypes associated with T cell activation, regulation, and exhaustion/inhibition (checkpoints). Concerning T-cell activation, overweight and obese patients had lower proportions of CD38^+^ cells than controls (all *p* < 0.0001). Only overweight patients had higher proportions of CD4^+^CD25^+^ T cells than controls (*p* < 0.05). Also, only patients with obesity had lower proportions of cytotoxic CD8 + T cells (CD38^+^GZB^+^) than controls (*p* < 0.05). No changes were noted for CD69^+^ or HLA-DR^+^ phenotypes. Contrasting changes were reported in CD25^+^CD127^low/neg^ regulatory T cells, with increased and decreased proportions found in CD4^+^ and CD8^+^ T cells, respectively (all *p* = 0.002). There were similar proportions of T cells expressing most exhaustion and/or checkpoint inhibitors across all groups, except for a slight increase in CD4^+^LAG-3^+^ T cells in patients with obesity (*p* < 0.05). Next, we investigated the expression of cell-surface markers by the analysis of the median fluorescence intensity (MFI), Table 3. T cells of COVID-19 patients showed downregulated expression of activation receptors (CD38 and HLA-DR) than controls. Checkpoint inhibitors PD-1 and LAG-3 were also found downregulated in CD4^+^ T cells only in COVID-19 patients than controls.

**Table 2:** Proportions of activated, regulatory, and inhibitory T-cell subsets. Group comparisons were performed by One-Way (ANOVA) or Kruskal-Wallis

Subsets	Controls *n* = 11	COVID-19					
		Overweight *n* = 22	Obese *n* = 28			*p*-value	
**CD3**^**+**^CD4^**+**^							
CD38^+^	27.8 (6.8–56.7)	8.36 (1.37–14.1)^A^		6.21 (1.3–14.1)^A^			**0.0001**
HLA-DR^+^	1.28 (0.5–4.9)	1.48 (0.4–7.7)		1.37 (0.5–38.8)			0.9285
CD69^+^	6.55 (0.1–77.5)	3.86 (1.5–7.3)		4.87 (1.1–40.2)			0.1527
CD25^+^	26.50 (17.5–51.9)	38.00 (26.4–56.1) ^A^		37.25 (7.0–56.5)			**0.0455**
CD38^+^HLA-DR^+^	3.17 (1.5–11.1)	2.47 (0.2–7.7)		2.42 (0.1–10.7)			0.6388
CD25^+^CD69^+^	1.45 (0.0–13.9)	1.17 (0.3–12.2)		1.71 (0.3–29.0)			0.3861
CD25^LOW^CD127+	9.13 (4.8–29.8)	22.30 (12.1–61.6) ^A^		19.65 (0.1–29.8)			**0.0340**
CD25^-^CD127^+^	83.0 (12.2–91.2)	65.60 (27.2–78.8)		63.10 (35.2–82.8)			0.7142
CD25^-^CD127^-^	2.40 (0.4–13.5)	1.31 (0.0–18.5)		4.41 (0.0–48.5)			0.4452
CD25^+^CD127^low/neg^	0.23 (0.0–43.5)	3.33 (0.0–39.10) ^A^		4.89 (0.0–49.5) ^A^			**0.0023**
PD-1^+^	4.74 (0.2–15.4)	7.37 (1.8–33.0)		8.34 (2.2–60.7)			0.1320
TIM-3^+^	1.13 (0.0–29.2)	1.53 (0.5–32.2)		2.49 (0.3–66.7)			0.6394
LAG-3^+^	0.20 (0.0–1.0)	0.50 (0.0–12.5) ^A^		0.60 (0.7–66.7) ^A^			**0.0430**
CTLA-4^+^	1.70 (0.2–60.8)	0.55 (0.0–2.8)		0.69 (0.1–66.7)			0.0580
**CD3** ^**+**^ **CD8** ^**+**^							
CD38^+^	14.30 (11.1–52.0)	9.85 (0.5–33.6) ^A^		7.93 (0.3–33.1) ^A^			**0.0136**
HLA-DR^+^	3.15 (0.0–17.9)	3.11 (0.2–13.4)		3.24 (0.8–38.6)			0.7557
CD69^+^	5.40 (0.3–28.2)	7.83 (3.9–23.4)		5.40 (0.3–28.2)			0.1203
CD25^+^	19.80 (12.3–47.8)	26.80 (15.3–50.4)		28.55 (8.4–79.9)			0.2889
CD38^+^HLA-DR^+^	3.63 (0.5–12.1)	2.20 (0.1–14.1)		1.51 (0.3–18.0)			0.2910
CD38^+^GZB^+^	11.60 (2.1–35.2)	7.79 (1.2–72.5)		9.28 (1.1–39.6) ^A^			**0.0326**
CD38^+^HLA-DR^+^GZB^+^	31.60 (5.1–77.6)	28.20 (0.0–98.5)		21.10 (2.3–80.0)			0.3230
CD25^+^CD69^+^	3.39 (0.0–10.1)	1.81 (0.3–6.6)		2.54 (0.4–33.7)			0.2578
CD25^LOW^CD127+	2.29 (0.2–7.1)	9.40 (4.5–36.5) ^A^		9.63 (2.3–38.3) ^A^			**0.0008**
CD25^-^CD127^+^	7.55 (1.1–14.2)	34.20 (8.9–59.3) ^A^		32.00 (9.6–58.1) ^A^			**0.0001**
CD25^-^CD127^-^	72.70 (26.4–92.1)	4.1 (0.0–17.9)		4.10 (0.0–17.9) ^A^			**0.0182**
CD25^+^CD127^low/neg^	9.81 (3.4–55.5)	4.64 (1.0–20.3) ^A^		4.48 (0.0–20.2) ^A^			**0.0024**
PD-1^+^	1.05 (0.1–21.7)	1.97 (0.1–55.2)		2.16 (0.3–87.0)			0.8971
TIM-3^+^	4.17 (0.1–71.2)	1.79 (0.3–22.7)		2.61 (0.2–31.0)			0.4060
LAG-3^+^	0.28 (0.0–2.9)	0.68 (0.5–7.4)		0.46 (0.0–20.7)			0.2748
CTLA-4^+^	0.60 (0.0–28.6)	0.35 (0.0–2.84)		0.63 (0.0–27.60)			0.1349

Data are presented as mean (min - max). **A** Denotes statistical difference compared to Controls (*p* < 0.05). **B** Denotes statistical difference compared to COVID-19 overweight (*p* < 0.05)

**Table 3:** Median fluorescence intensity (MFI) of activated, senescence, and inhibitory checkpoints expressed by circulating T and NK cells. Group comparisons were performed by One-Way (ANOVA) or Kruskal-Wallis

Subsets	Controls *n* = 11	COVID-19				
		Overweight *n* = 22	Obese *n* = 28	*P*-value		
**CD3** ^**+**^						
CD3^+^	1744 (1199–2444)	1580 (1134–2548)	1570 (1049–2686)	0.3920		
CD38^+^	1023 (637–1573)	471.5 (258–903) ^A^	511.0 (165–951) ^A^	**0.0001**		
HLA-DR^+^	3356 (1309–7709)	951.0 (71.4–3261) ^A^	1070 (62.8–6944) ^A^	**0.0001**		
CD25^+^	254.0 (233–1754)	528.0 (264–1523)	752 (358–2181)	0.2710		
CD57^+^	0.60 (0.0–28.6)	0.60 (0.0–28.6)	0.60 (0.0–28.6)	0.6880		
NKG2A^+^	770.0 (180–13,318)	590.5 (410–7946)	551.0 (300–2291)	0.7930		
NKG2D^+^	983.0 (738–242,647)	875.5 (625–1212)	782.5 (692–2654) ^A^	**0.0282**		
**CD3** ^**+**^ **CD4** ^**+**^						
CD27^+^	2990 (1351–4919)	5437 (2214–7652) ^A^	5348 (2951–6922) ^A^	**0.0001**		
CD28^+^	3126 (2159–6627)	594.5 (339–2103) ^A^	615.0 (256–1158) ^A^	**0.0001**		
CD57^+^	190.0 (143–4662)	9252 (337–52,204) ^A^	12,476 (164–46,083) ^A^	**0.0003**		
NKG2D^+^	869 (627–1048)	821 (650–4919)	837 (658–10,422)	0.2850		
CD45RA^+^	655.0 (332–14,299)	1696 (917–8246)	1783 (1207–4200)	0.4070		
PD-1^+^	928.0 (103–3366)	96.5 (69.3–2670) ^A^	97.30 (69.3-10423) ^A^	**0.0033**		
TIM-3^+^	1699 (1248–3289)	1413 (1101–3969)	1572 (1101–4123)	0.6190		
LAG-3^+^	710.0 (552–1646)	609 (306–1518)	529.5 (335–1585) ^A^	**0.0429**		
CTLA-4^+^	703.0 (452–2984)	550.5 (397–4048)	179.0 (397–4048)	0.6380		
**CD3** ^**+**^ **CD8** ^**+**^						
CD27^+^	2122 (780–4794)	5250 (359–8048) ^A^	5326 (573–6922) ^A^	**0.0002**		
CD28^+^	2215 (1790–2545)	351.0 (198–1025) ^A^	375.5 (164–715) ^A^	**0.0001**		
CD57^+^	4976 (812–21,010)	16,619 (5007–32,721) ^A^	12,512 (2355–32,406) ^A^	**0.0035**		
NKG2A^+^	1585 (682–4097)	1267 (781–15,112)	1091 (793–2807)	0.7100		
NKG2D^+^	1107 (789–3362)	1099 (1072–1839)	1130 (987–1334)	0.5440		
CD45RA^+^	891.0 (332–12,824)	3260 (1003–25,702)	3282 (575–8213)	0.7930		
PD-1^+^	1114 (58.6–3366)	686.5 (0.0–39,226)	1596 (271–26,750)	0.5930		
TIM-3^+^	2104 (1699–3289)	1960 (1601–2781)	2052 (1457–5111)	0.3120		
LAG-3^+^	687.0 (0.0–1235)	624.5 (481–1696)	615.0 (0.0–1920)	0.9200		
CTLA-4^+^	966.0 (776–2984)	1214 (0.0–8405)	1238 (0.0–3851)	0.7120		
**CD3** ^**-**^ **CD56** ^**+**^						
CD57^+^	1049 (490–3808)	1900 (512–25,580)	1297 (366–7663)	0.3440		
NKG2A^+^	3990 (724–11,952)	1369 (640–11,698)	1400 (989–11,563) ^A^	**0.0178**		
NKG2D^+^	1062 (788–2818)	1040 (782–2612)	1162 (673–2819)	0.8390		
PD-1^+^	666.0 (541–974)	673.0 (510–4080)	622.5 (487–1256)	0.8650		

Data are presented as mean (min - max). **A** Denotes statistical difference compared to Controls (*p* < 0.05). **B** Denotes statistical difference compared to COVID-19 overweight (*p* < 0.05)

### Expansion of T cells with senescence profile in COVID-19

**Fig. 4:**
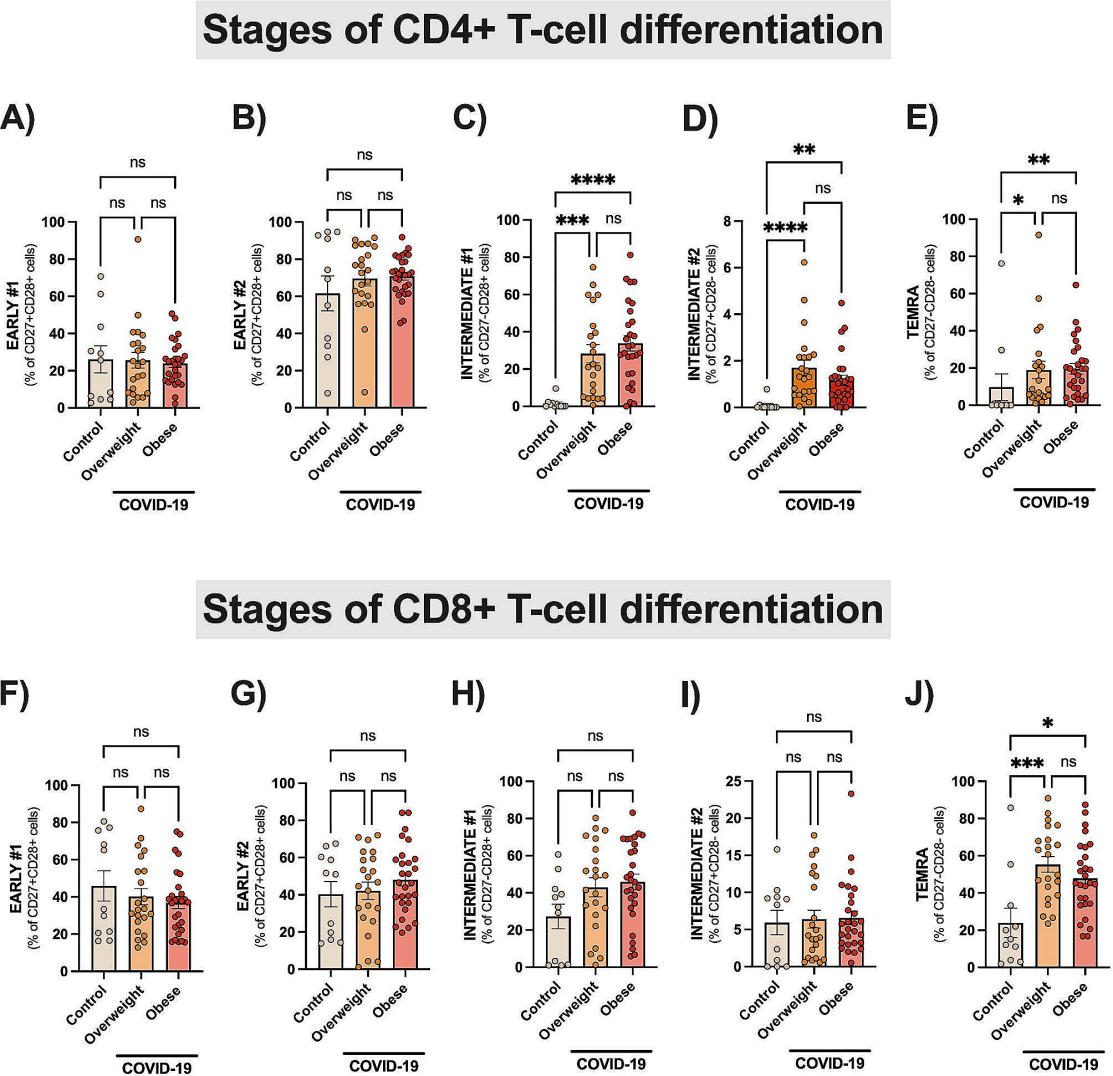
Different stages of T-cell differentiation. Data are shown as mean ± SE and scatter plots. Early differentiated T cells were defined by the expression of CD4+/CD8 + and CD27 + CD28 + CD57-CD45RA+ (early #1) or CD45RA- (early #2). Intermediate-differentiated T cells were defined by the expression of CD4+/CD8 + and CD27-CD28 + CD57 + CD45RA- (intermediate #1) or CD27 + CD28-CD57 + CD45RA- (intermediate #2). TEMRA cells were defined by the expression of CD4+/CD8 + and CD27-CD28-CD45RA + CD57+. Statistically significant differences are indicated: * *p* < 0.05, ** *p* < 0.001 and **** *p* < 0.0001 (Kruskal-Wallis, Dunn’s multiple comparison test)

**Fig. 5:**
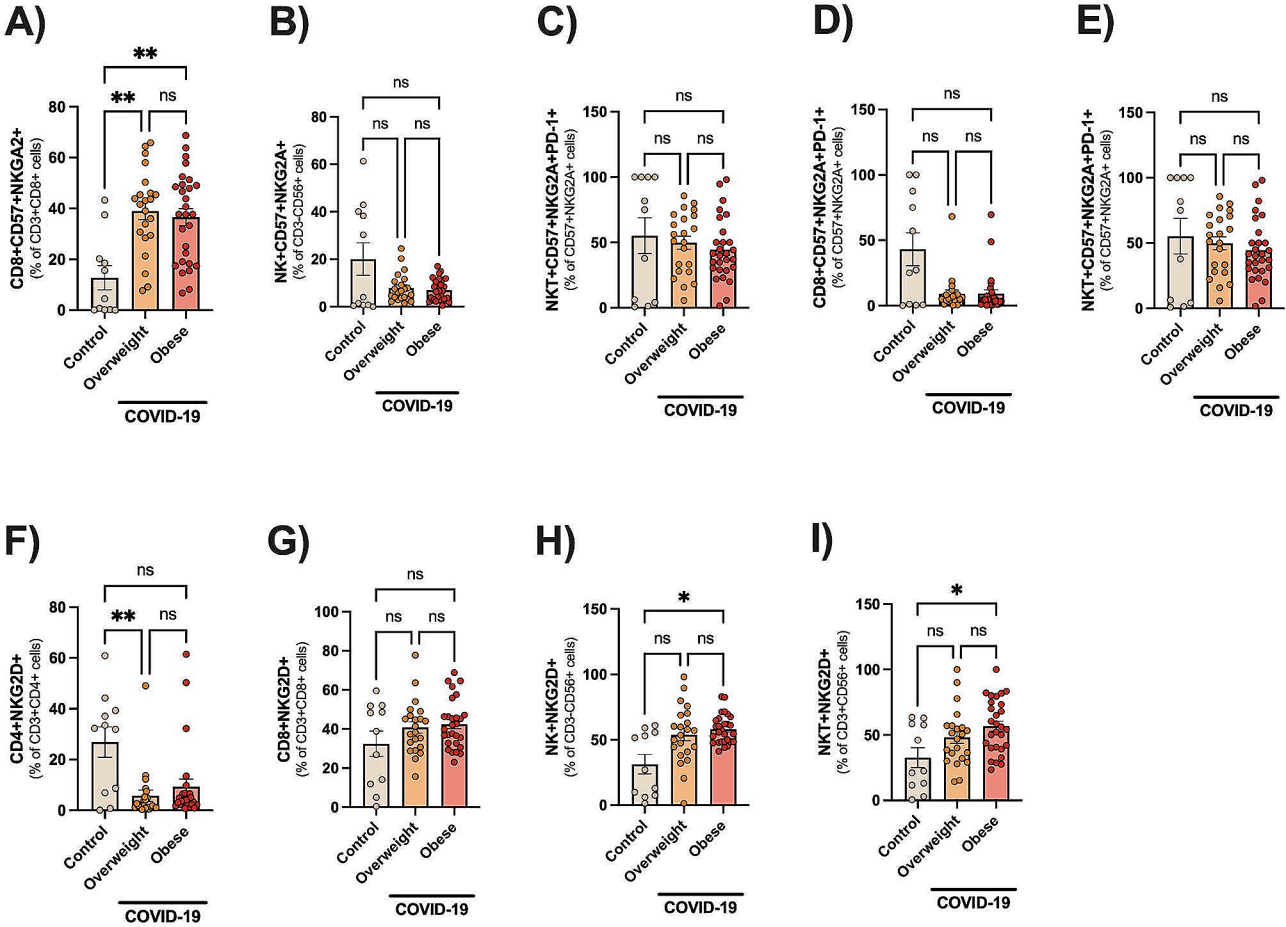
Expansion of T cells with senescent profiles in hospitalized patients with COVID-19. Data are shown as mean ± SE and scatter plots. Statistically significant differences are indicated: * *p* < 0.05, and ** *p* < 0.01 (Kruskal-Wallis, Dunn’s multiple comparison test)

Different stages of T-cell differentiation can be determined based on the cell-surface expression of the costimulatory molecules CD27, CD28, CD57 and CD45RA [25]. As shown in Fig. 4, the intermediate-differentiated CD4 + T cells (CD27^−^CD28^+^CD57^+^CD45RA^−^) and CD4 + or CD8 + TEMRA cells (CD27^−^CD28^−^CD57^+^CD45RA^+^) were found to be greatly expanded in patients than in controls (all *p* < 0.001). The CD27 expression was found upregulated in both T-cell subsets of COVID-19 patients than controls (∼ 2-fold), Table 3. In contrast, there was a robust downregulation of CD28 in T-cell subsets (∼ 5-fold) of patients than controls (Table 3). Also, the CD57 (senescent marker) was found greatly upregulated in T-cell subsets of COVID-19 patients, with prominent increased expression in CD4^+^ T cells (∼ 57-fold) as compared to CD8^+^ T cells (∼ 3-fold), Table 3. T-cell senescence can be also described by the expression of NK receptors (NKG2A/D) [26, 27], allowing aged T cells to kill altered/stressed cells in an antigen-independent manner. COVID-19 Patients had a robust expansion of CD8^+^CD57^+^NKG2A^+^ T cells than controls (all *p* < 0.001), Fig. 5A.

**Fig. 6:**
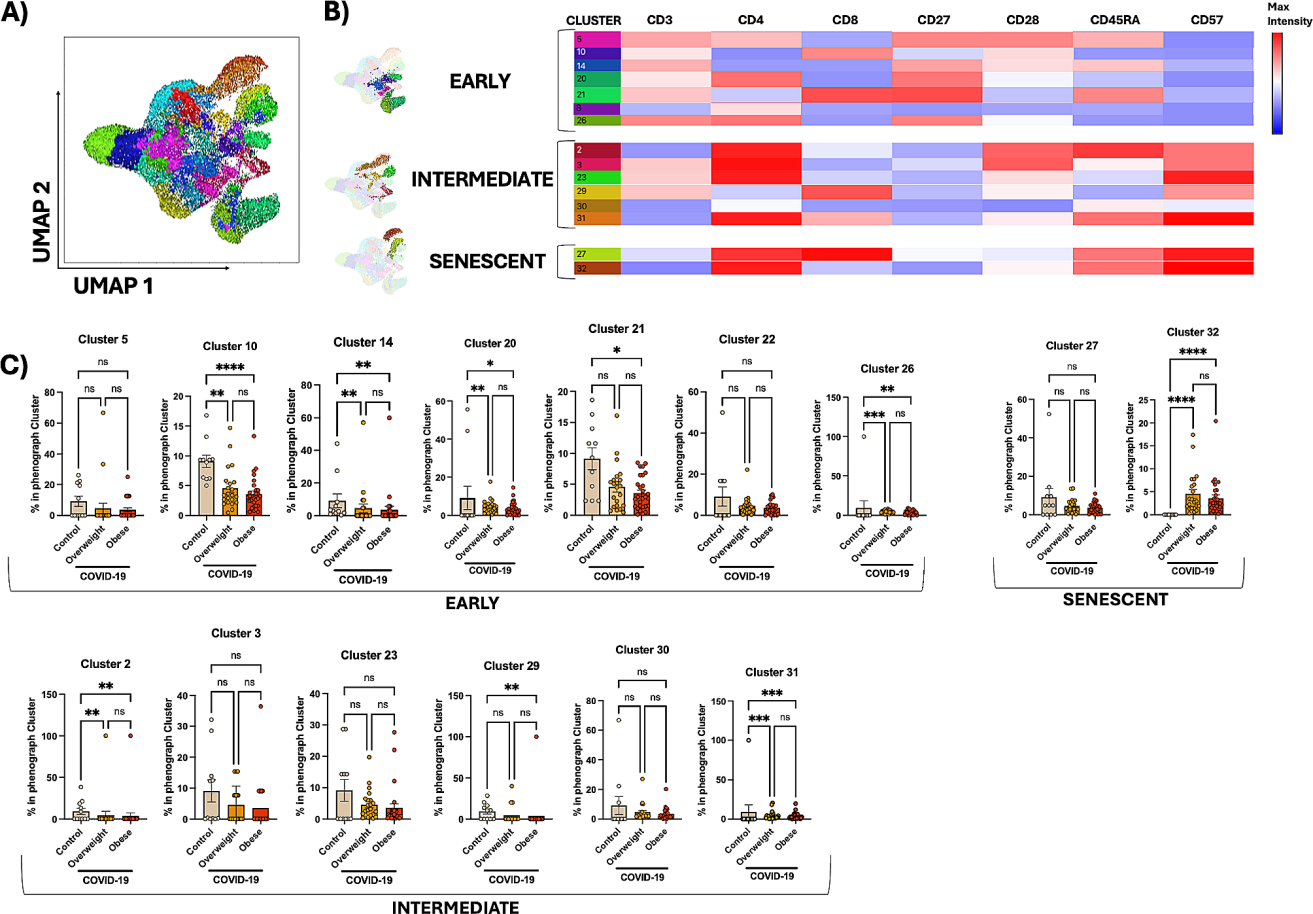
Unbiased immune profiling of the T-cell differentiation confirms the expansion of senescent clusters in COVID-19. (**A**) The Uniform Manifold Approximation and Projection (UMAP) method was performed in PBMCs (panel #3, stages of T-cell differentiation) to reduce dimensionality and 32 meta clusters were selected based on the expression of CD3, CD4, CD8, CD27, CD28, CD57, and CD45RA molecules. (**B**) Heatmap displaying the relative expression (representativeness) of selected 15 clusters classified into different stages of T-cell differentiation (early, intermediate, and senescent/TEMRA). Additionally, 17 clusters were characterized by increased single expression of CD27, CD28, and CD4, as well as non-T cells and unspecified (see Figure S3). Shades of red represent increased expression while shades of blue denote decreased expression for each indicated marker. (**C**) The percentage of each cluster in Phenograph-identified clusters among the studied groups (control, overweight, and obese). Statistically significant differences are indicated: * *p* < 0.05, ** *p* < 0.01 and **** *p* < 0.0001 (Kruskal-Wallis, Dunn’s multiple comparison test)

We next sought to further investigate T-cell differentiation and senescence phenotypes by unbiased immune profiling and clustering techniques (Fig. 6, S3 and S4). UMAP was performed to reduce dimensionality in PBMCs of panel #3 (stages of T-cell differentiation) and 32 clusters were selected based on the expression of CD3, CD4, CD8, CD27, CD28, CD57, and CD45RA molecules (Figure S3). The percentage of each cluster in UMAP is shown in Figure S3C. To get a deep insight into relevant lymphocytes, 15 clusters were classified into different stages of T-cell differentiation (early, intermediate, and senescent/TEMRA) based on the expression of CD3, CD4, CD8, CD27, CD28, CD57, and CD45RA molecules (Fig. 6B). Figure 6 C shows the percentage of each cluster in Phenograph-identified clusters (i.e., representativeness) among the studied groups and confirms the expansions of senescent T cells in COVID-19 (cluster 32). In addition, 17 clusters were characterized by increased single expression of CD27, CD28, and CD4, as well as non-T cells and unspecified (Figure S3). Similarly, UMAP was performed to reduce dimensionality in PBMCs of panel #6 (senescence) and 31 clusters were selected based on the expression of CD3, CD8, CD56, CD57, PD-1, and NKG2A (Figure S4A, S4B and S4C). Figure S4D shows the major lymphocyte subsets identified in the UMAP. Figure S4E shows the percentage of each cluster in Phenograph-identified clusters among the studied groups. Several clusters had reduced representativeness in COVID-19 patients, like total T cells (ex., clusters 1, 5, 6). In contrast, patients had increased proportions of Phenograph-identified clusters of CD3^low^ T cells expressing high levels of CD57 and NKG2A than controls (cluster 31).

### Associations with sociodemographic and clinical parameters

The hospitalized COVID-19 groups were homogenous on sociodemographic (age, sex, and ethnicity) and clinical parameters assessed (mean length of hospital admission, ICU admission, mortality rates). Nonetheless, we sought to explore the potential impact of these parameters on the immune readouts. Concerning mortality, COVID-19 non-survivors (8.9 ± 2.69) had reduced proportions of CD3^+^CD4^+^ T cells than survivors (17.8 ± 8.99), *p* = 0.01. No differences or associations were found for the remaining variables. Regarding ICU admission, no differences were found between subjects admitted and those not admitted to the ICU. Regarding sex-related changes, females had slightly higher adiponectin levels (2,684.54 ± 234.69) than males (2,487.56 ± 236.35), *p* = 0.01. No differences or associations were found for the remaining variables (including age). In COVID-19 patients, BMI was found positively correlated with activated CD4^+^CD69^+^ T cells (Rho = 0.58, *p* < 0.05), and NK cells (Rho = 0.41, *p* = 0.003) as well as negatively correlated with adiponectin levels (Rho= -0.31, *p* < 0.05).

## Discussion

Hospitalized patients with COVID-19 have several dysfunctional innate and adaptive immune responses associated with clinical progression. Obesity may affect how the immune system responds to COVID-19, especially in older adults. Here, we report that hospitalised obese/overweight COVID-19 patients are associated with increased markers of microbial translocation, dysfunctional innate mononuclear cells, and pronounced expansion of late-differentiated or senescent T cells.

First, we confirm previous evidence indicating that COVID-19 is associated with increased markers of bacterial translocation (i.e., LPS and sCD14) [12, 13]. Disruption in gut barriers and microbial translocation could result from SARS-CoV-2 intestine infection or hyperactive host innate cells, releasing pro-inflammatory cytokines that alter lung/gut permeability [33]. Severe COVID-19 patients presenting higher LPS levels also report a higher number of gastrointestinal symptoms, including nausea, vomiting and diarrhoea [34]. Increased gut permeability would lead to increased leakage of gut commensals and associated metabolites into circulation, contributing to the exacerbation of systemic inflammation by activating innate immune cells, such as neutrophils and monocytes [35]. LPS is derived from gram-negative bacteria and is a potent stimulus for activating innate immune cells (e.g., monocytes) expressing Toll-like receptor 4 (TLR-4) [13]. Plasma levels of sCD14 have been established as a strong marker of monocyte activation [36]. Interestingly, increased LPS and sCD14 were associated with increased expression of NF-κB in CD14^+^CD16^+^ monocytes [34]. Also, endotoxemia initiates obesity and insulin resistance [37] as well as fuelling metabolic inflammation [38]. Therefore, obesity and COVID-19 may synergise with a leaky gut, contributing to hyperactivated innate immune responses. New therapies aimed to reduce the leaky gut would be helpful in COVID-19. The COVID-19 therapeutic benefits of microbiota-targeted interventions may include faecal microbiota transplantation (FMT), bacteriotherapy and traditional Chinese medicine (TCM) [39].

Several innate immunological pathways have been implicated in the COVID-19 pathogenesis [40], including impaired type I IFN levels and overactive responses (ex., activated monocytes, cytokine storm, neutrophil extracellular traps or NETs). Both ageing and obesity are associated with overactive innate immune responses including activated monocytes, neutrophils (with increased NETs), and activated inflammasome, contributing to chronic low-grade inflammation (“inflammaging”), thrombotic events, and the need for anticoagulants [41–43]. Macrophages play a central role in the development of COVID-19, and pro-inflammatory monocyte-derived macrophages are abundant in bronchoalveolar lavage (BAL) fluids from patients with critical COVID-19 [40]. Here, hospitalised COVID-19 patients had significant expansions of classical monocytes. The expansion of classical monocytes could be involved with immunopathology and disease progression as previous studies demonstrated the role of classical monocytes in driving the hyper-inflammatory phenotype in severe COVID-19 [44, 45]. Hospitalized COVID-19 patients had also reduced frequencies of intermediate monocytes, in line with previous work [46]. However, there are discrepancies in the literature concerning the fluctuations of monocyte phenotypes in COVID-19 [13, 47], and this could be interpreted according to the differences in clinical severity and viral load between studies.

Investigating the role of NK cells in COVID-19 is crucial, as they play a key role in antiviral immunity. Our study revealed distinct NK cell profiles in COVID-19 patients compared to healthy controls. Notably, patients exhibited a similarly larger expansion of immature NK cells (CD56^+^CD16^−^) alongside a reduction in mature NK cells (CD56^+^CD16^+^). This is significant because mature NK cells possess greater cytotoxic potential and cytokine production (IFN-γ, TNF-α) upon activation compared to their immature counterparts [48]. Our findings are in line with previous studies showing reduced counts of mature NK cells, of note in critically ill patients [49, 50]. Lower counts of NK cells were associated with impaired anti-SARS-CoV-2 NK cell activity, which was particularly prominent and prolonged in severe COVID-19 [51]. Similar findings were reported in uninfected obese subjects. Indeed, subjects with obesity show reduced proportions of peripheral NK cells compared with lean controls, and these NK cells are dysfunctional: producing less IFN-γ, expressing lower levels of GZB and perforin, as well as reduced cytotoxicity towards tumour target cells [52]. Next, we examined different NK cell phenotypes involved with cellular activation (NKG2D), inhibition (NKG2A) and NK cell maturity (CD57). COVID-19 was associated with expansions of NK and NKT cells expressing the activating receptor NKG2D. However, no changes were noted for the phenotypes expressing the inhibitory receptor NKG2A or CD57. Ageing is generally associated with increased counts/proportions of circulating NK cells, albeit impaired cytotoxicity [53, 54].

Lymphopenia, widely documented in acute viral infections including SARS-CoV-2, was confirmed here as indicated by the reduced proportions of T cells. Lymphopenia has been predictive of poor disease outcomes in COVID-19, and is a risk factor for secondary infections, accounting for 50% of estimated mortality secondary to COVID-19 [55, 56]. In contrast, COVID-19 patients with obesity and overweight had a similar prominent expansion (3-fold) of plasmablasts than controls. Plasmablasts are precursors of plasma cells and are known for their ability to secrete large amounts of antibodies. Indeed, significant expansions of plasmablasts reaching up to 30% circulating B cells have previously been reported in severe COVID-19 patients [9, 16, 57]. Plasmablasts are proliferating cells, highly metabolically active and it was speculated they could modulate immune responses in COVID-19 by acting as a nutrient sink [57]. However, few studies have studied these cells in the context of obesity, and plasmablasts were reported significantly to be increased in patients with rheumatoid arthritis with BMI > 25 [58]. Obesity may accelerate age-related defects in human B cells. As a potential mechanism involved, we report that COVID-19 patients with obesity had higher leptin levels than controls. Leptin, an adipokine secreted primarily by adipocytes, has been associated with intrinsic B cell inflammation and immunosenescence, impaired generation of protective antibodies, as well as increased generation of autoimmune antibodies [59] – supporting the concept that obesity accelerates immunosenescence. Obesity may also dampen the immunity to various pathogens, particularly influenza, translating to a heightened risk of hospitalizations and deaths from the seasonal flu [60]. In this way, leptin impairs the antiviral type 1 interferon response against influenza and highlights the negative impact of obesity on the immune response.

Several studies reported that, while numerically decreased, both CD4^+^ and CD8^+^ T cells from severe COVID-19 patients present dysregulated features of increased activation and exhaustion [61]. To gain a deeper insight into these features in patients with overweight/obesity, several T-cell phenotypes were characterized concerning activation, regulation, and inhibition/exhaustion profiles. COVID-19 patients had lower proportions of activated CD38^+^ T cells, whereas obese patients had reduced percentages of cytotoxic CD8 + T cells (CD38^+^GZB^+^) than controls. CD25, CD38, and CD69 molecules are crucial early-activation T-cell markers that have been previously associated with severe disease outcomes in COVID-19 patients. Given the significant impact of obesity on immune cells and the association of obesity with more severe COVID-19 outcomes, it is reasonable to speculate that T-cells from overweight or COVID-19 patients with obesity, could be considered dysfunctional for effective anti-viral responses. Concerning Treg cells, patients had opposite changes for the CD25^+^CD127^low/neg^ phenotype: increased proportions were observed in the CD4 + whilst reduced percentages in the CD8 + T cells. This is in line with a previous study reporting an increased percentage of CD4^+^CD25^+^CD127^low^ Treg in patients with mild COVID-19 during hospitalization compared to during the recovery period [62]. It should be noted, however, that severe COVID-19 was associated with reduced proportions of CD4^+^CD25^+^CD127^low^ Treg cells [63]. Furthermore, COVID-19 patients, of note critically ill subjects, have more dysfunctional T cells associated with exhaustion and inhibition (PD-1, TIM-3, LAG-3, CTLA-4, and NKG2A) [14, 64]. Here, except for a marginal increase in CD4 + LAG-3 + cells, there were no changes in exhaustion and/or checkpoint inhibitors between groups.

Finally, we sought to investigate whether accelerated immunosenescence was established in hospitalized subjects with COVID-19 and excess of body weight. COVID-19 patients had significant expansions of more differentiated T cells, including TEMRA cells. This was observed with greater magnitude among CD8^+^ than CD4^+^ T cells. Next, premature senescence was also confirmed by a prominent expansion of CD8^+^CD57^+^NKG2A^+^ T cells in COVID-19 patients than controls. The expression of NK receptors on T cells is a feature of human senescence [26, 27], allowing aged T cells to kill altered/stressed cells in an antigen-independent manner. Finally, unbiased immune profiling further confirmed the expansions of senescent T cells in COVID-19. Expansions of highly differentiated or senescent T cells (CD27^−^CD28^−^) have been reported in COVID-19, of note in severe cases [65–68]. The expansion of more differentiated T cells could be a consequence of constant SARS-CoV-2 stimulation as well as related to obesity. Recent data provides evidence that mediators found in the plasma of subjects with obesity are likely to promote premature immunosenescence. Healthy PBMCs exposed to plasma from individuals with obesity showed an increase in the pool of late-differentiated CD8^+^CD28 T cells. Also, PBMCs from obese individuals had shortened telomeres, indicating replicative senescence [69, 70]. Senescent T cells acquire cytotoxic features, including the expression of NK receptors, as well as developing the senescence-associated secretory phenotype (SASP) [26]. SASP factors include several pro-inflammatory cytokines, chemokines, proteases, and growth factors that can contribute to the ‘cytokine storm’, tissue-destructive immune cell infiltration, endotheliitis, fibrosis and micro thrombosis – all of which are important for COVID-19 pathophysiology and clinical progression. The expansion of late-differentiated CD28^null^ cells in COVID-19 could be also related to cytomegalovirus (CMV) co-infection, as CMV is considered one of the main factors driving accelerated immunosenescence [71]. Although CMV serology was not investigated here, a recent study reports that CD28^null^ T cells were only found expanded in CMV + nonhospitalized adults with mild COVID-19 [72]. In addition, higher expansions of SARS-CoV-2 reactive TEMRA cells (CD8^+^) were noted in CMV + unexposed young subjects as compared with CMV- subjects [73]. This could be explained by the fact that pre-existing T-cell immunity to SARS-CoV-2 involves T cells recognizing common viral antigens like influenza and CMV [74].

Our data should be interpreted considering some limitations. One of the primary limitations is the absence of eutrophic individuals with COVID-19, which hinders a comprehensive assessment of how weight status influences the course of COVID-19 and associated immune responses. Therefore, the findings of our study should be interpreted with the understanding that they may not fully capture the complete spectrum of COVID-19 outcomes across different weight categories. This limitation underscores the need for future research to include a more diverse representation of weight and clinical categorisations to better understand the relationship between excess weight, immune function, and COVID-19 severity. Despite this limitation, our study observed significant differences in most of the studied phenotypes with the same magnitude and direction in both overweight and patients with obesity. Moreover, the patients enrolled in our study were controlled for various sociodemographic and clinical variables that could have influenced the results, mitigating potential confounding factors and strengthening the reliability of the observed associations. However, it is important to note that the lack of CMV serology and data on absolute counts of studied subsets may have limited the scope of our study. Another limitation is the heterogeneity of COVID-19 vaccination among subjects, which may have influenced the observed immune responses. Additionally, the sample size of the control group was relatively small, and we cannot exclude the possibility of type II errors. Despite these limitations, our study provides valuable insights into the complex interplay between weight status, immune function, and COVID-19 status, highlighting the need for further research to expand upon and validate our findings.

## Conclusions

Our study reveals that COVID-19 patients with excess of body weight exhibit higher plasma levels of LPS and sCD14, as well as expansions of dysfunctional innate and adaptive immune cells, lower T-cell activation, and expansion of senescent cells.

### Electronic supplementary material

Below is the link to the electronic supplementary material.

Supplementary Material 1

Supplementary Material 2

Supplementary Material 3

**Supplementary Material 4: Figure S3.** Unbiased immune profiling of T-cell differentiation panel. (A) The Uniform Manifold Approximation and Projection (UMAP) method was performed in PBMCs (panel #3, stages of T-cell differentiation) to reduce dimensionality and 32 meta clusters were selected based on the expression of CD3, CD4, CD8, CD27, CD28, CD57, and CD45RA molecules. (B) Heatmap displaying the relative expression (representativeness) of selected 15 clusters classified into different stages of T-cell differentiation (early, intermediate, and senescent/TEMRA). Additionally, 17 clusters were characterized by increased single expression of CD27, CD28, and CD4, as well as non-T cells and unspecified (see Figure S3). Shades of red represent increased expression while shades of blue denote decreased expression for each indicated marker. (C) The percentage of each cluster in Phenograph-identified clusters among the studied groups (control, overweight, and obese). Statistically significant differences are indicated: * *p* < 0.05, ** *p* < 0.01 and **** *p* < 0.0001 (Kruskal-Wallis, Dunn’s multiple comparison test)

**Supplementary Material 5: Figure S4.** Unbiased immune profiling of senescent panel. (A) The Uniform Manifold Approximation and Projection (UMAP) analysis led us to identify 31 meta clusters based on the expression of CD3, CD8, CD56, CD57, PD-1, and NKG2A molecules. (B) Heatmap displaying the relative expression (representativeness) of 31 clusters. Shades of red represent increased expression while shades of blue denote decreased expression for each indicated marker. (C) The proportion of each cluster in UMAP. (D) The 31 clusters were separated according to the indicated subsets. (E) The percentage of each cluster in Phenograph-identified clusters among the studied groups (control, overweight, and obese). Statistically significant differences are indicated: * *p* < 0.05, ** *p* < 0.01 and **** *p* < 0.0001 (Kruskal-Wallis, Dunn’s multiple comparison test)

## Data Availability

No datasets were generated or analysed during the current study.
