# Supplementary material for: Excess of body weight is associated with accelerated T-cell senescence in hospitalized COVID-19 patients
Source: Immun Ageing. 2024 Mar 8;21:17. doi: 10.1186/s12979-024-00423-6 (PMC10921685; doi:10.1186/s12979-024-00423-6)

MONOCYTES

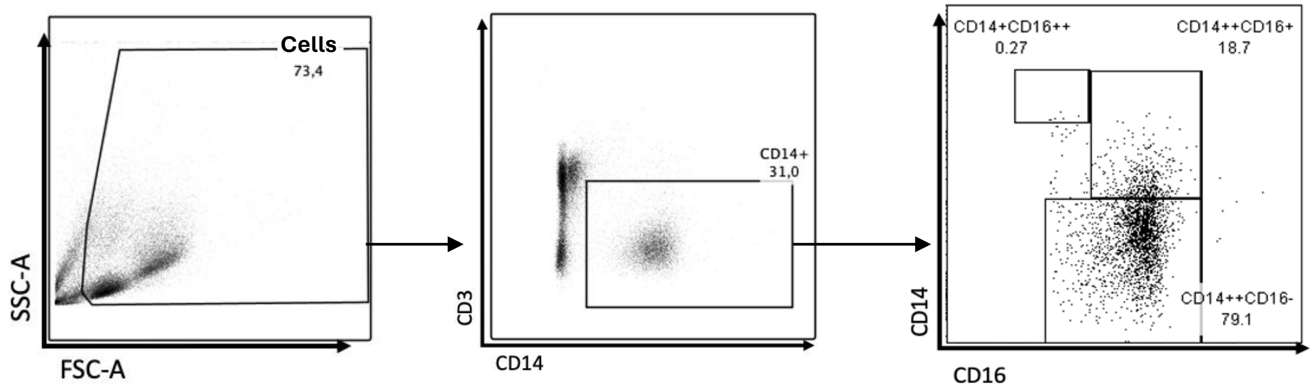

NK and NKT

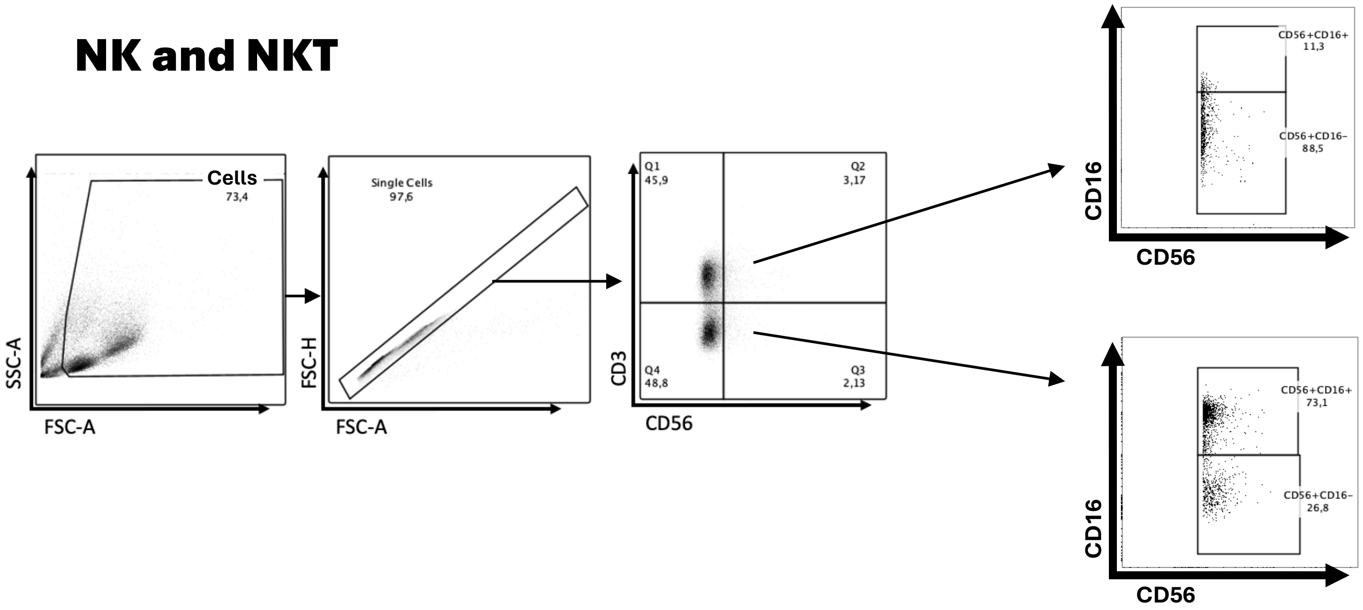

B CELLS

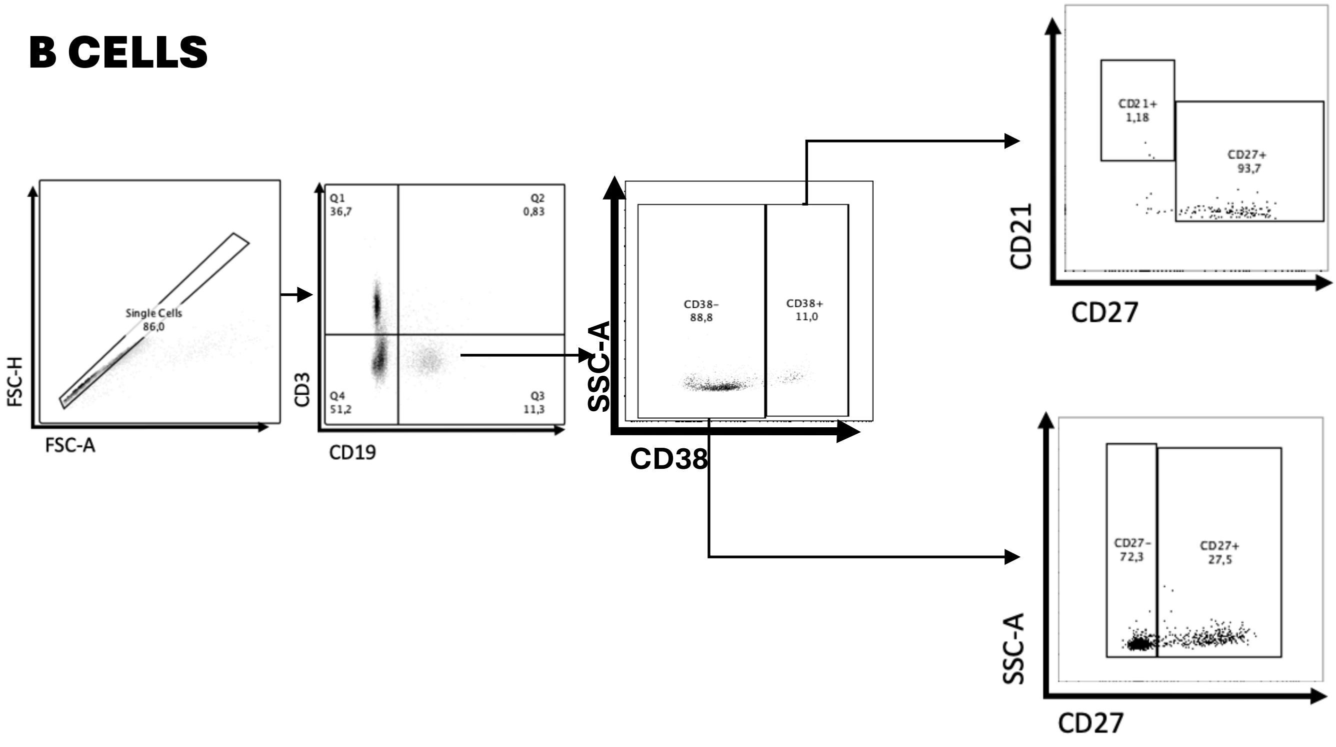

T CELLS

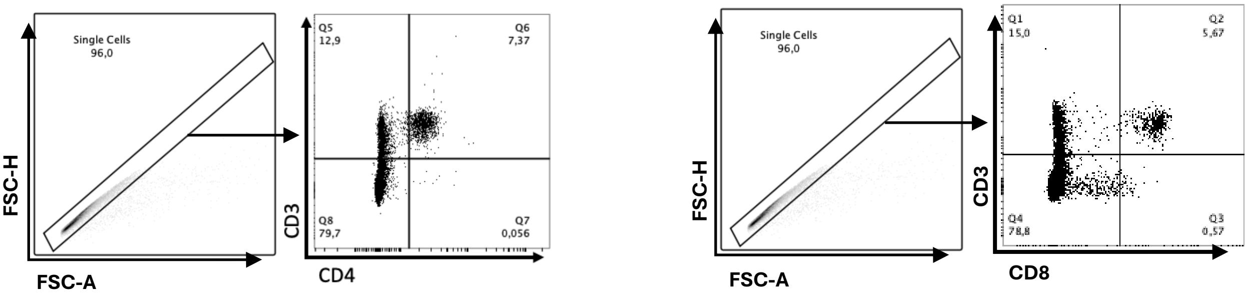

T-CELL DIFFERENTIATION STAGES

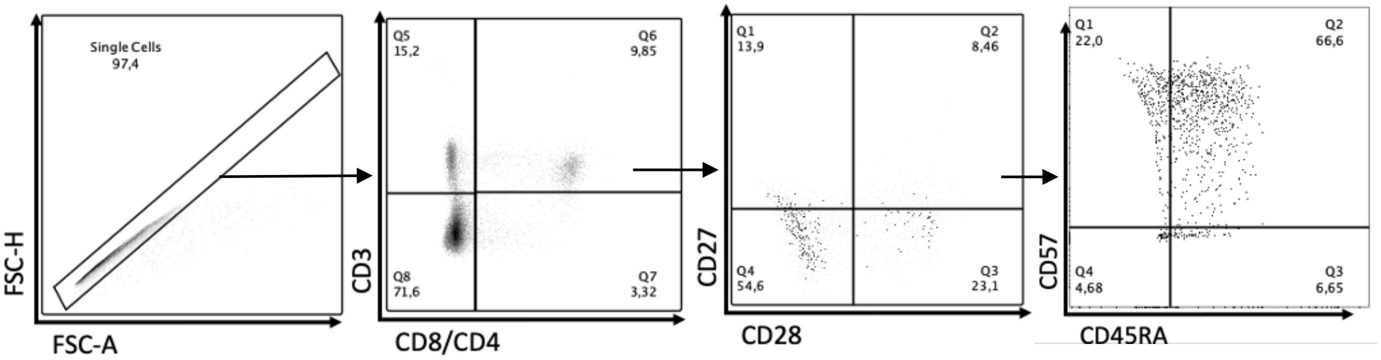

ACTIVATED AND CYTOTOXIC T CELLS

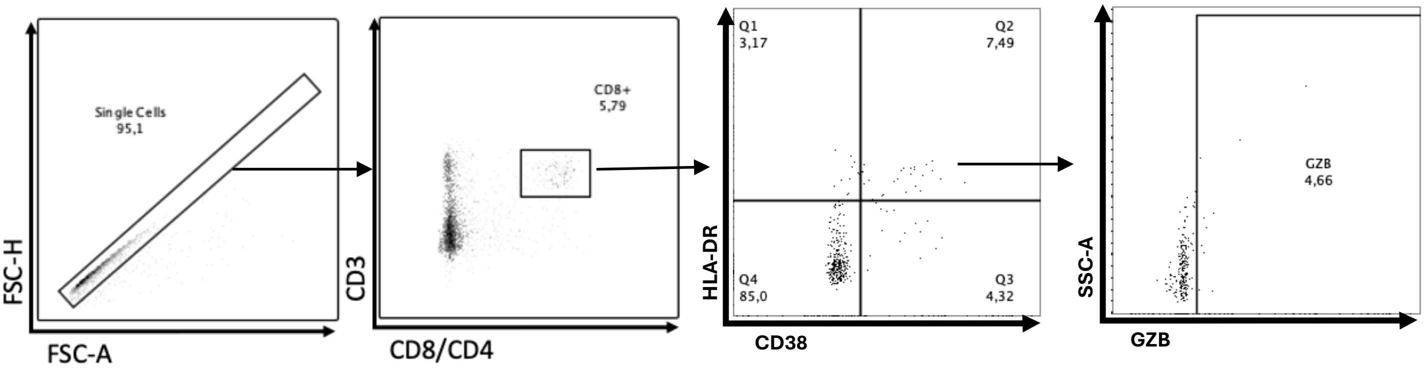

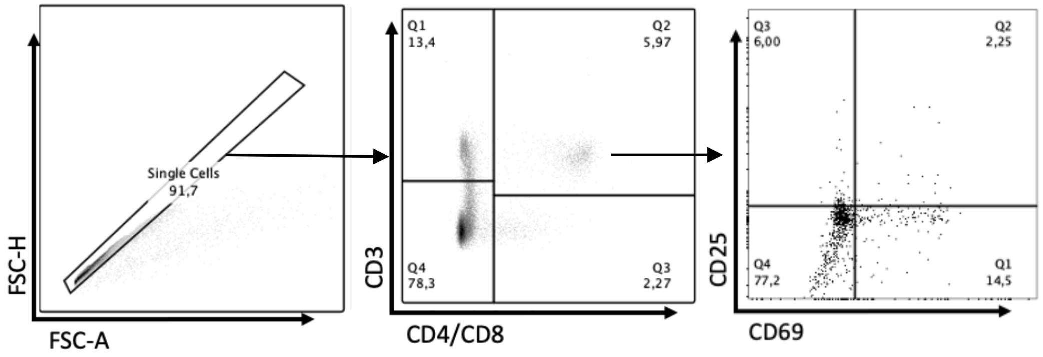

REGULATORY T CELLS

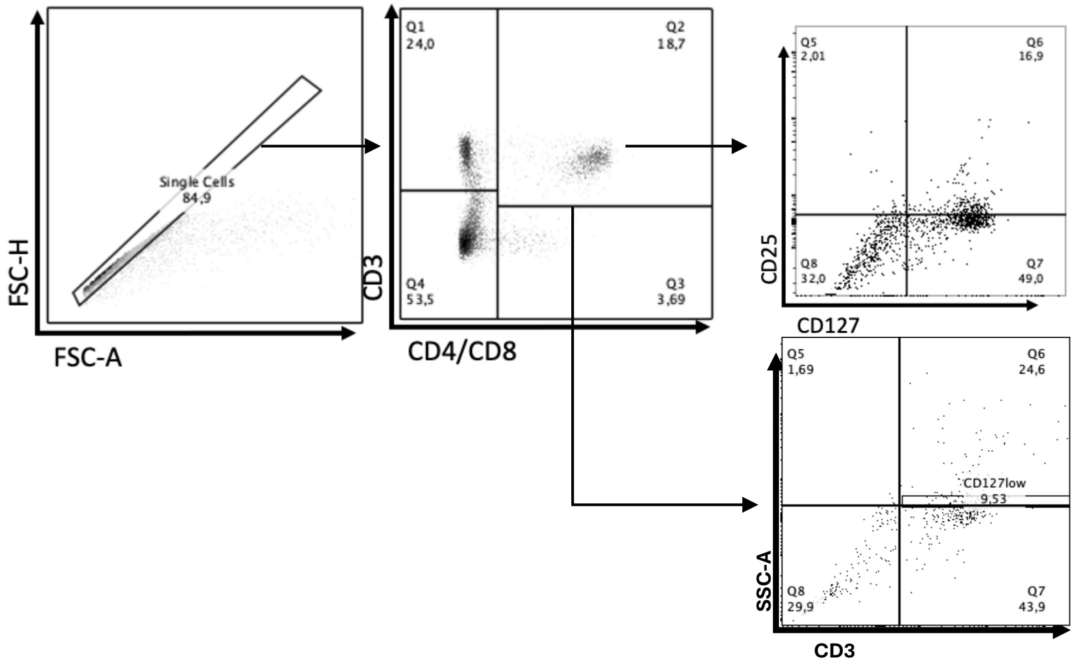

CHECKPOINTS (Inhibition, Exhaustion, Apoptosis)

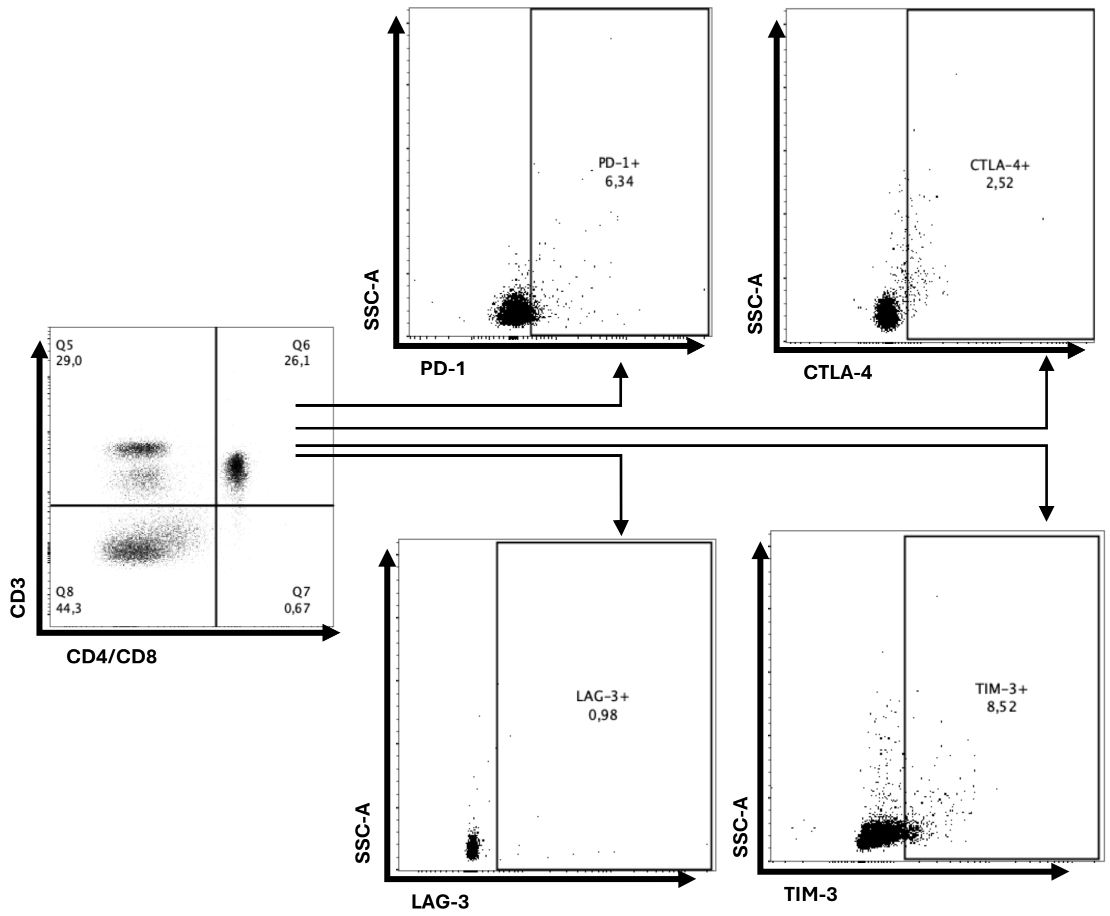

NKG2A, CD57 AND PD-1

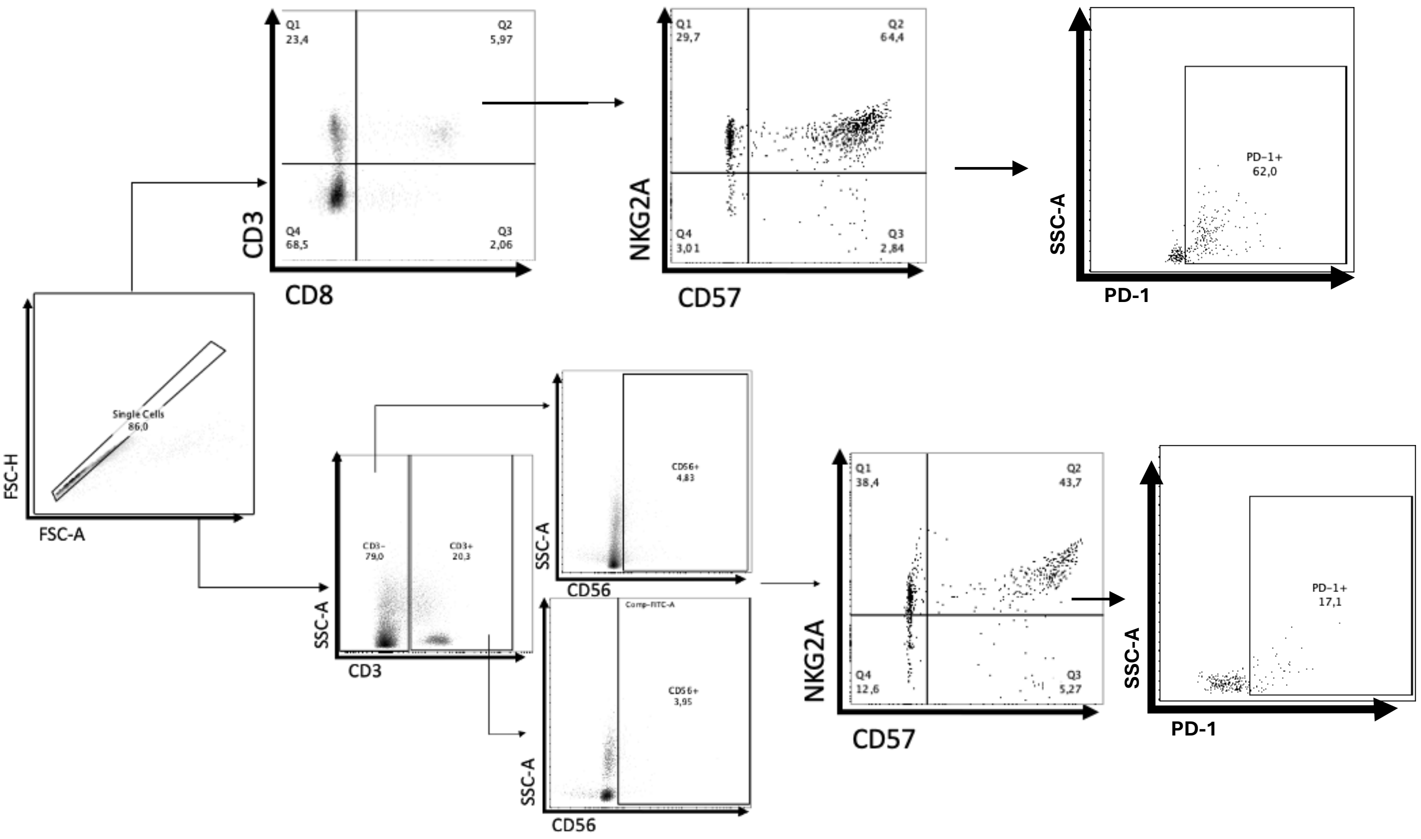

NKG2D ON T CELLS and NK

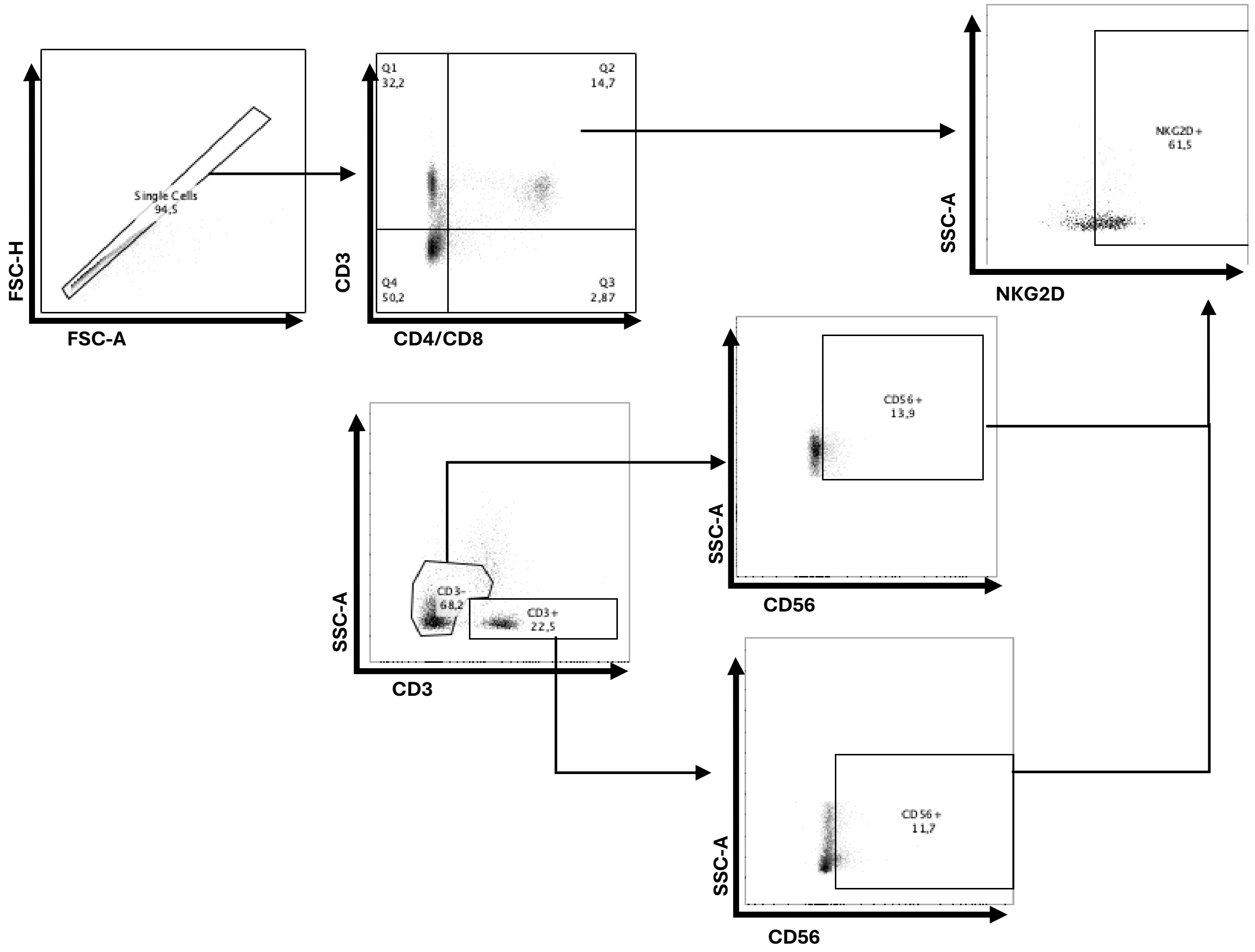

Supplement: Supplementary file 2 — Supplementary Material 2 [file 12979_2024_423_MOESM2_ESM.pdf]
