# Supplementary material for: Excess of body weight is associated with accelerated T-cell senescence in hospitalized COVID-19 patients
Source: Immun Ageing. 2024 Mar 8;21:17. doi: 10.1186/s12979-024-00423-6 (PMC10921685; doi:10.1186/s12979-024-00423-6)
Supplement: Supplementary file 3 — Supplementary Material 3 [file 12979_2024_423_MOESM3_ESM.pdf]

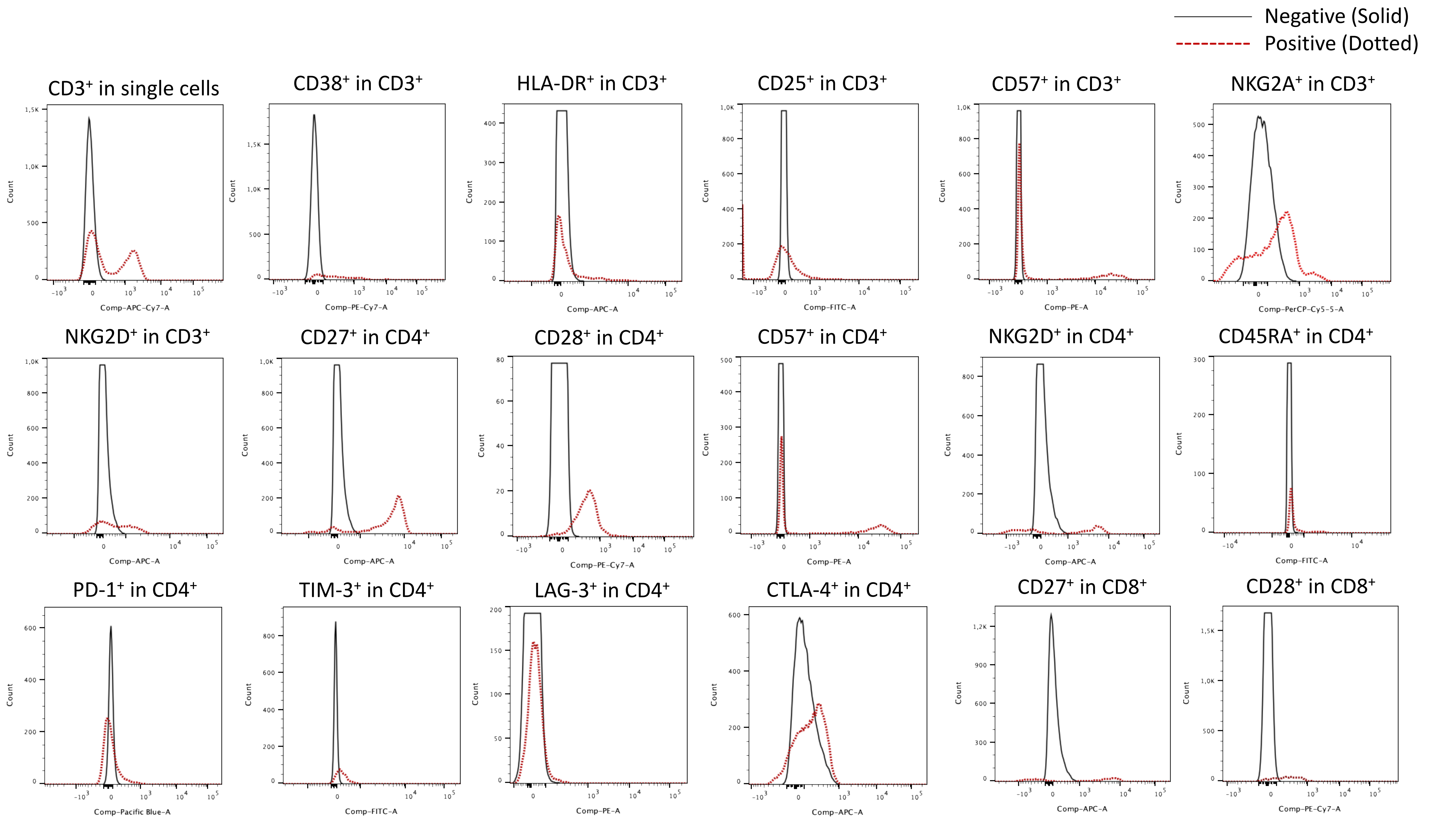

Negative (Solid)
Positive (Dotted)
CD3$^+$ in single cells
Comp-APC-Cy7-A
CD38$^+$ in CD3$^+$
Comp-PE-Cy7-A
HLA-DR$^+$ in CD3$^+$
Comp-APC-A
CD25$^+$ in CD3$^+$
Comp-FITC-A
CD57$^+$ in CD3$^+$
Comp-PE-A
NKG2A$^+$ in CD3$^+$
Comp-PerCP-Cy5-5-A
NKG2D$^+$ in CD3$^+$
Comp-APC-A
CD27$^+$ in CD4$^+$
Comp-APC-A
CD28$^+$ in CD4$^+$
Comp-PE-Cy7-A
CD57$^+$ in CD4$^+$
Comp-PE-A
NKG2D$^+$ in CD4$^+$
Comp-APC-A
CD45RA$^+$ in CD4$^+$
Comp-FITC-A
PD-1$^+$ in CD4$^+$
Comp-Pacific Blue-A
TIM-3$^+$ in CD4$^+$
Comp-FITC-A
LAG-3$^+$ in CD4$^+$
Comp-PE-A
CTLA-4$^+$ in CD4$^+$
Comp-APC-A
CD27$^+$ in CD8$^+$
Comp-APC-A
CD28$^+$ in CD8$^+$
Comp-PE-Cy7-A
Count

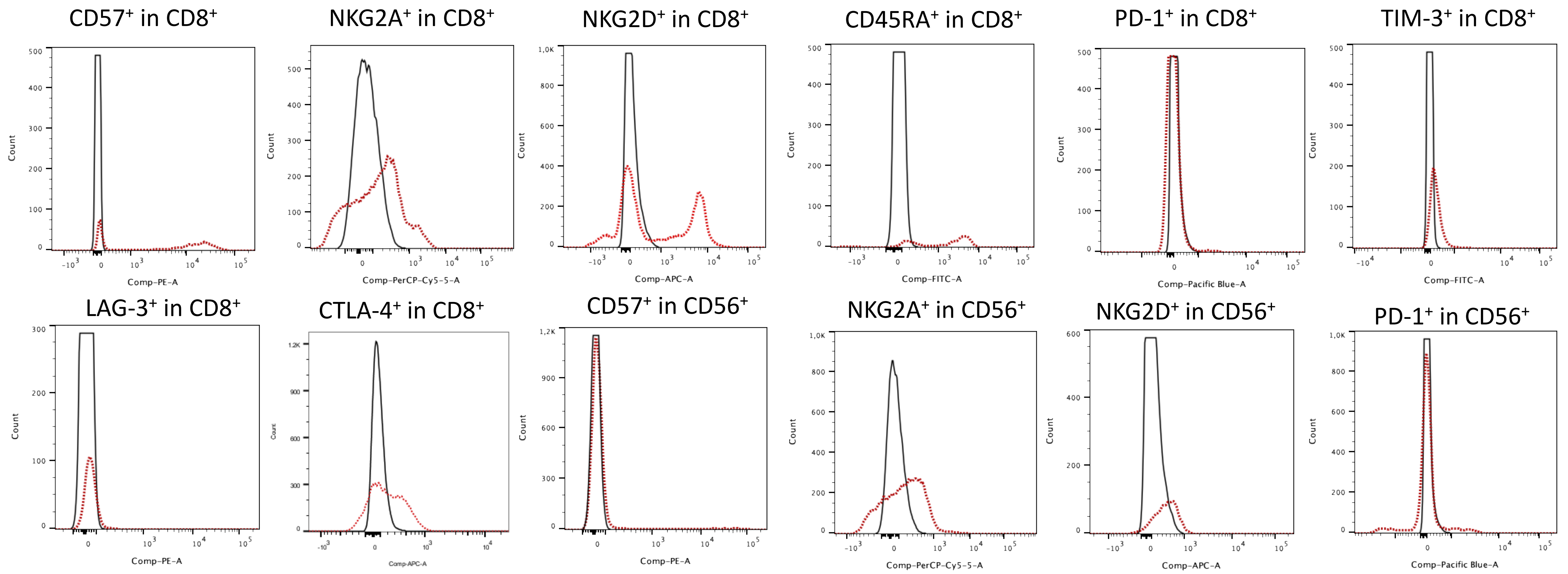
CD57⁺ in CD8⁺
Count
Comp-PE-A
NKG2A⁺ in CD8⁺
Count
Comp-PerCP-Cy5-5-A
NKG2D⁺ in CD8⁺
Count
Comp-APC-A
CD45RA⁺ in CD8⁺
Count
Comp-FITC-A
PD-1⁺ in CD8⁺
Count
Comp-Pacific Blue-A
TIM-3⁺ in CD8⁺
Count
Comp-FITC-A
LAG-3⁺ in CD8⁺
Count
Comp-PE-A
CTLA-4⁺ in CD8⁺
Count
Comp-APC-A
CD57⁺ in CD56⁺
Count
Comp-PE-A
NKG2A⁺ in CD56⁺
Count
Comp-PerCP-Cy5-5-A
NKG2D⁺ in CD56⁺
Count
Comp-APC-A
PD-1⁺ in CD56⁺
Count
Comp-Pacific Blue-A
