# Supplementary figures and images for: Excess of body weight is associated with accelerated T-cell senescence in hospitalized COVID-19 patients
Source: Immun Ageing. 2024 Mar 8;21:17. doi: 10.1186/s12979-024-00423-6 (PMC10921685; doi:10.1186/s12979-024-00423-6)

A)

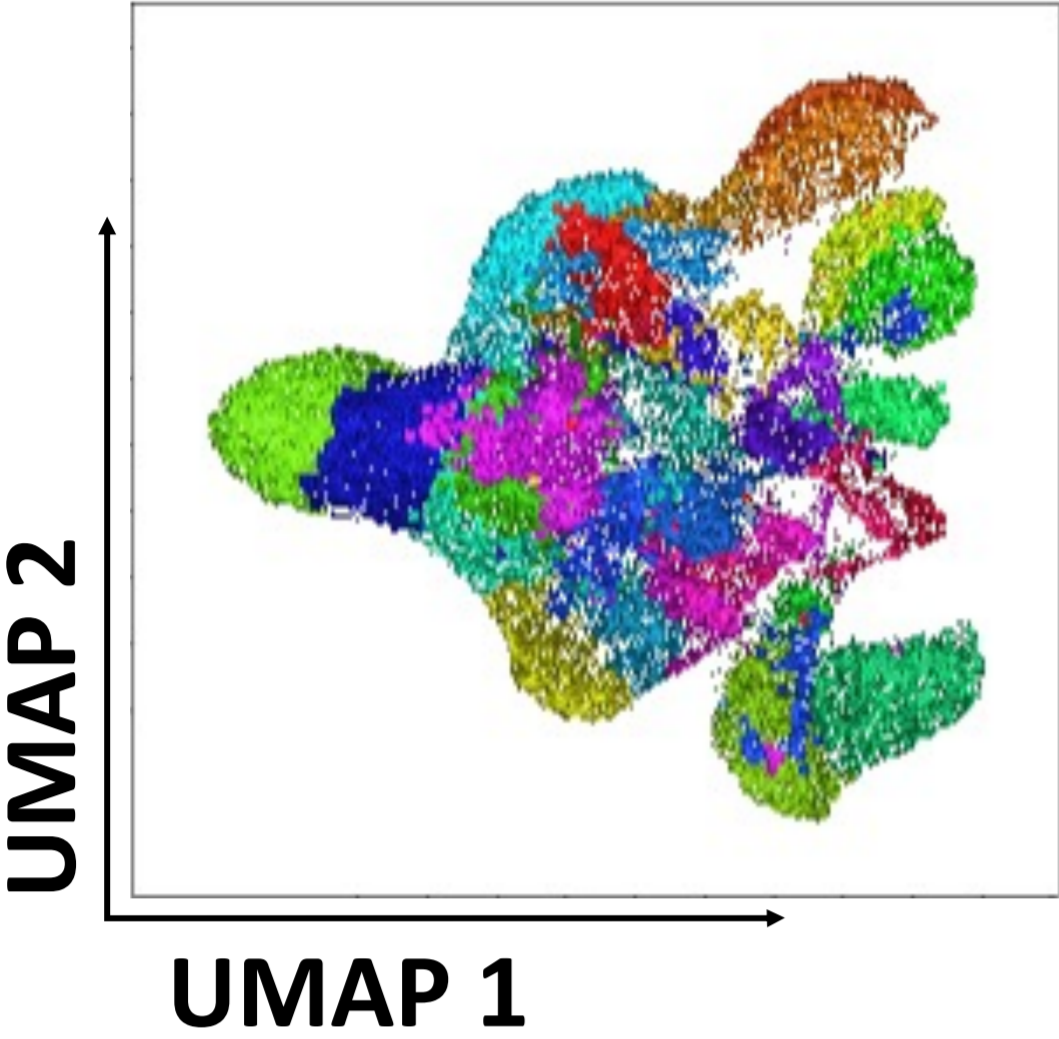

C)

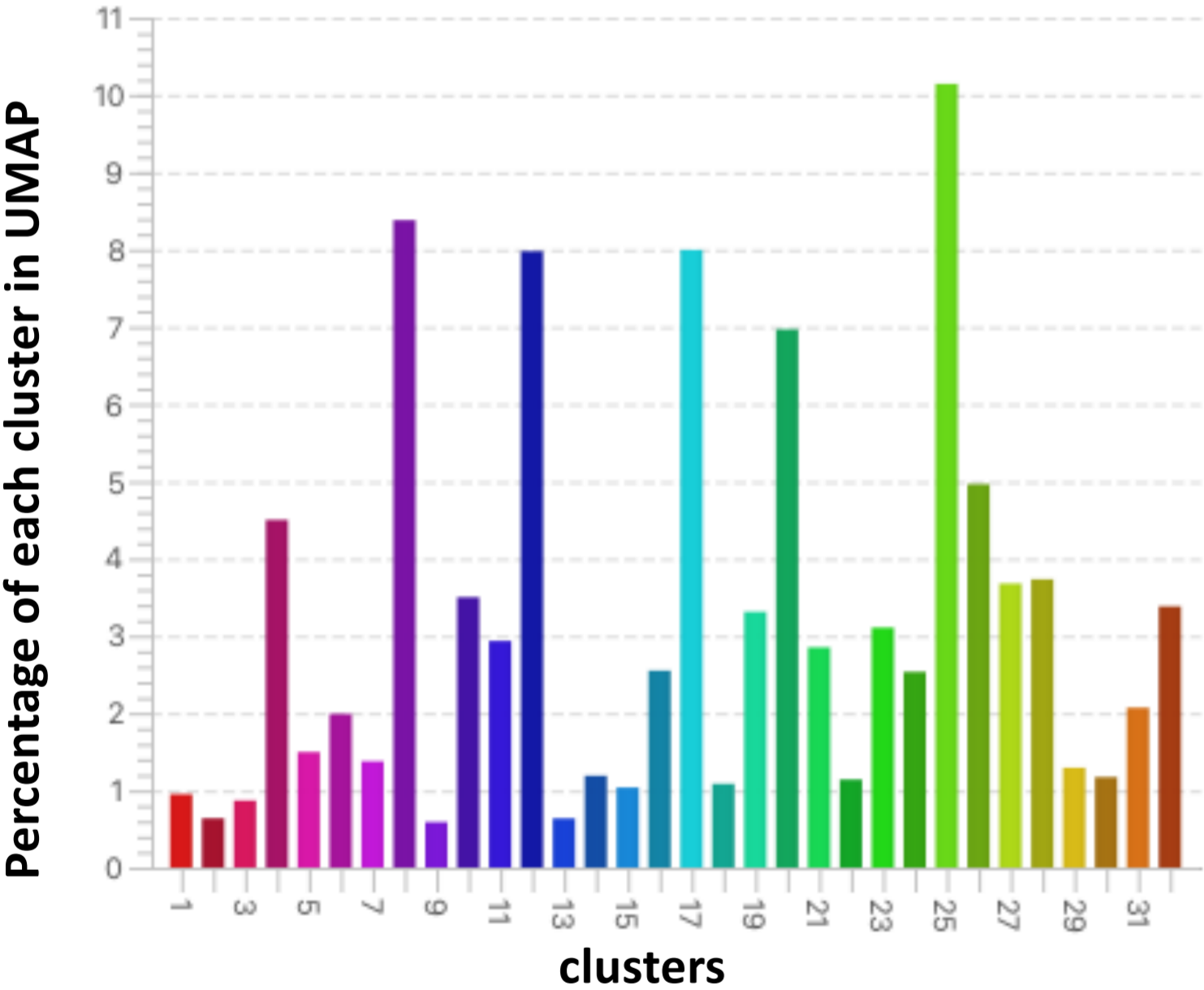

B)

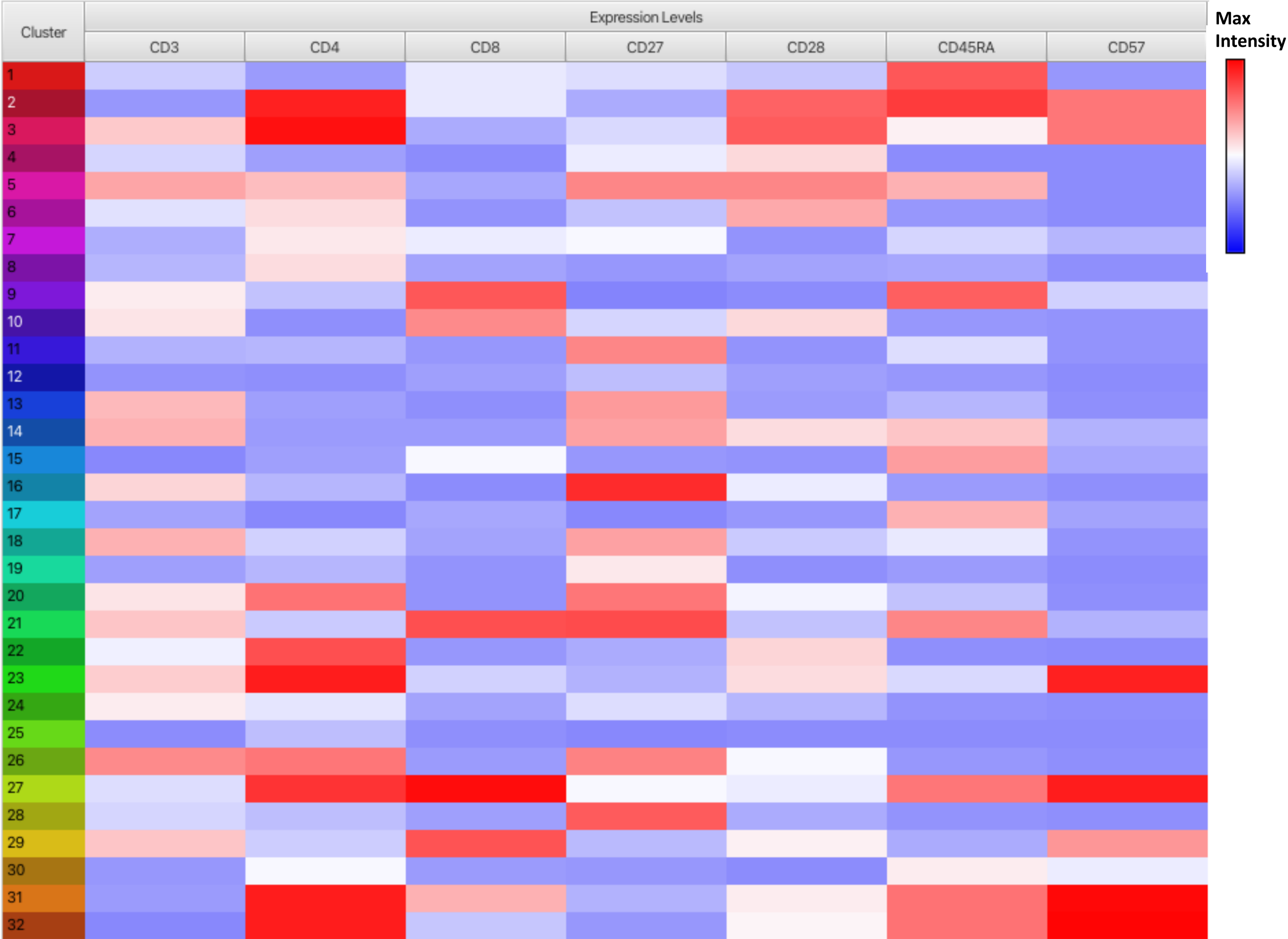

D)

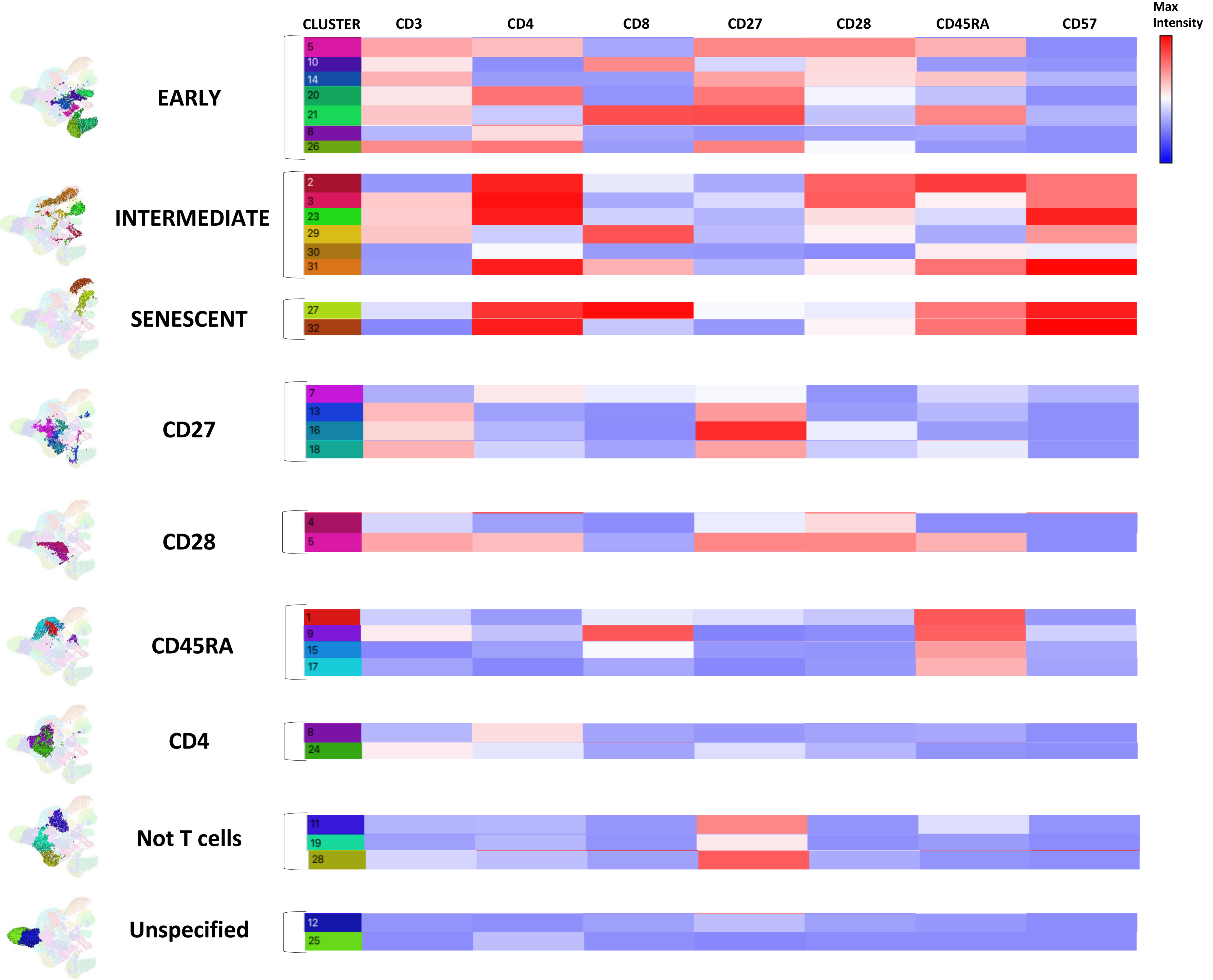

E)

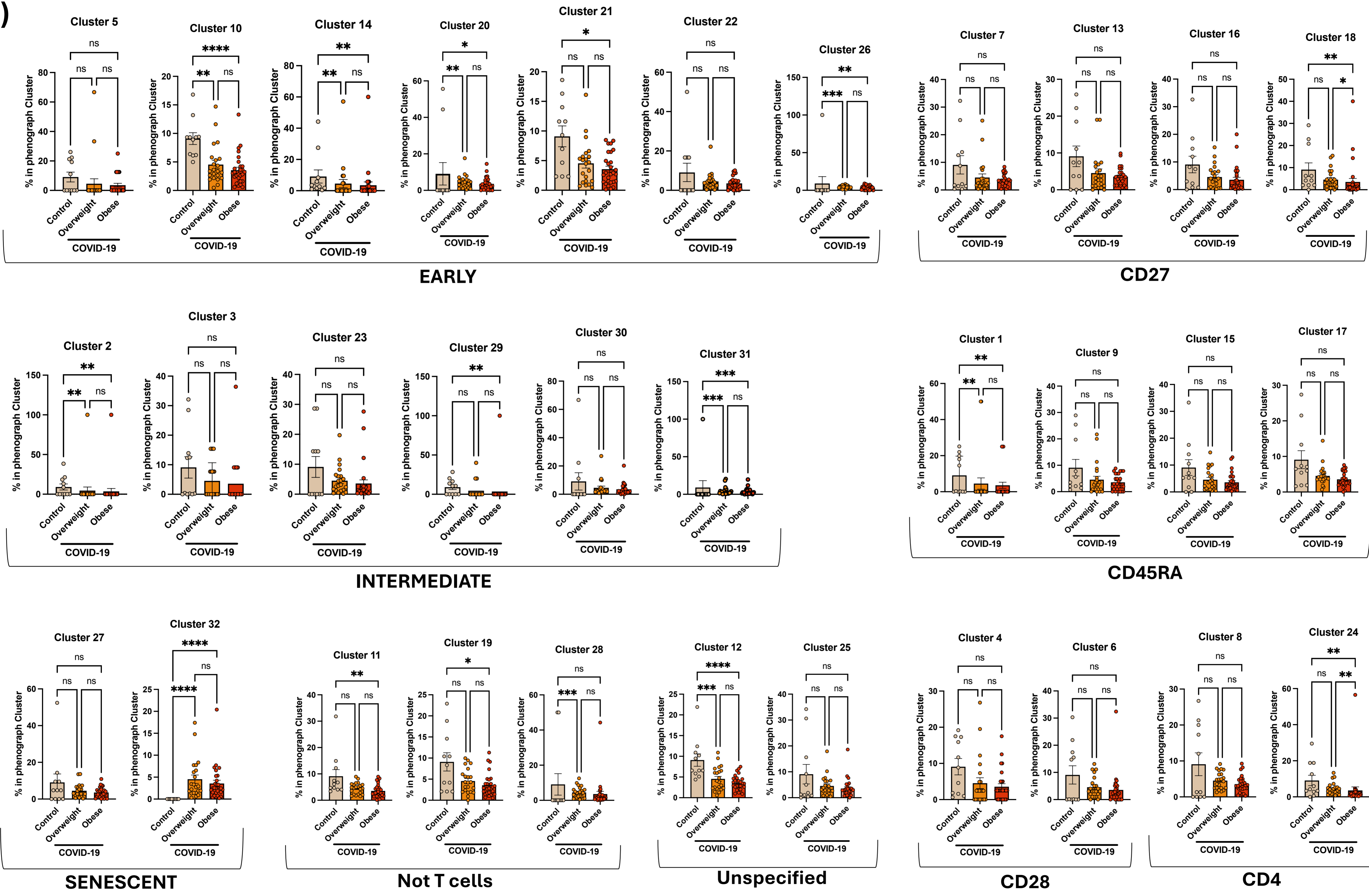

Supplement: Supplementary file 4 — Supplementary Material 4: Figure S3. Unbiased immune profiling of T-cell differentiation panel. (A) The Uniform Manifold Approximation and Projection (UMAP) method was performed in PBMCs (panel #3, stages of T-cell differentiation) to reduce dimensionality and 32 meta clusters were selected based on the expression of CD3, CD4, CD8, CD27, CD28, CD57, and CD45RA molecules. (B) Heatmap displaying the relative expression (representativeness) of selected 15 clusters classified into different stages of T-cell differentiation (early, intermediate, and senescent/TEMRA). Additionally, 17 clusters were characterized by increased single expression of CD27, CD28, and CD4, as well as non-T cells and unspecified (see Figure S3). Shades of red represent increased expression while shades of blue denote decreased expression for each indicated marker. (C) The percentage of each cluster in Phenograph-identified clusters among the studied groups (control, overweight, and obese). Statistically significant differences are indicated: * p < 0.05, ** p < 0.01 and **** p < 0.0001 (Kruskal-Wallis, Dunn’s multiple comparison test) [file 12979_2024_423_MOESM4_ESM.pdf]

A)

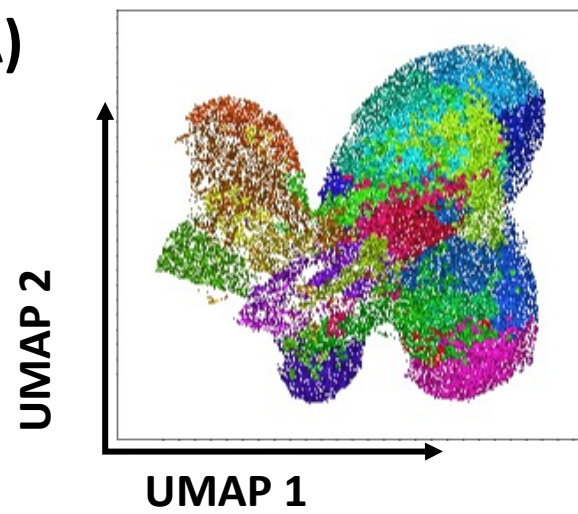

B)

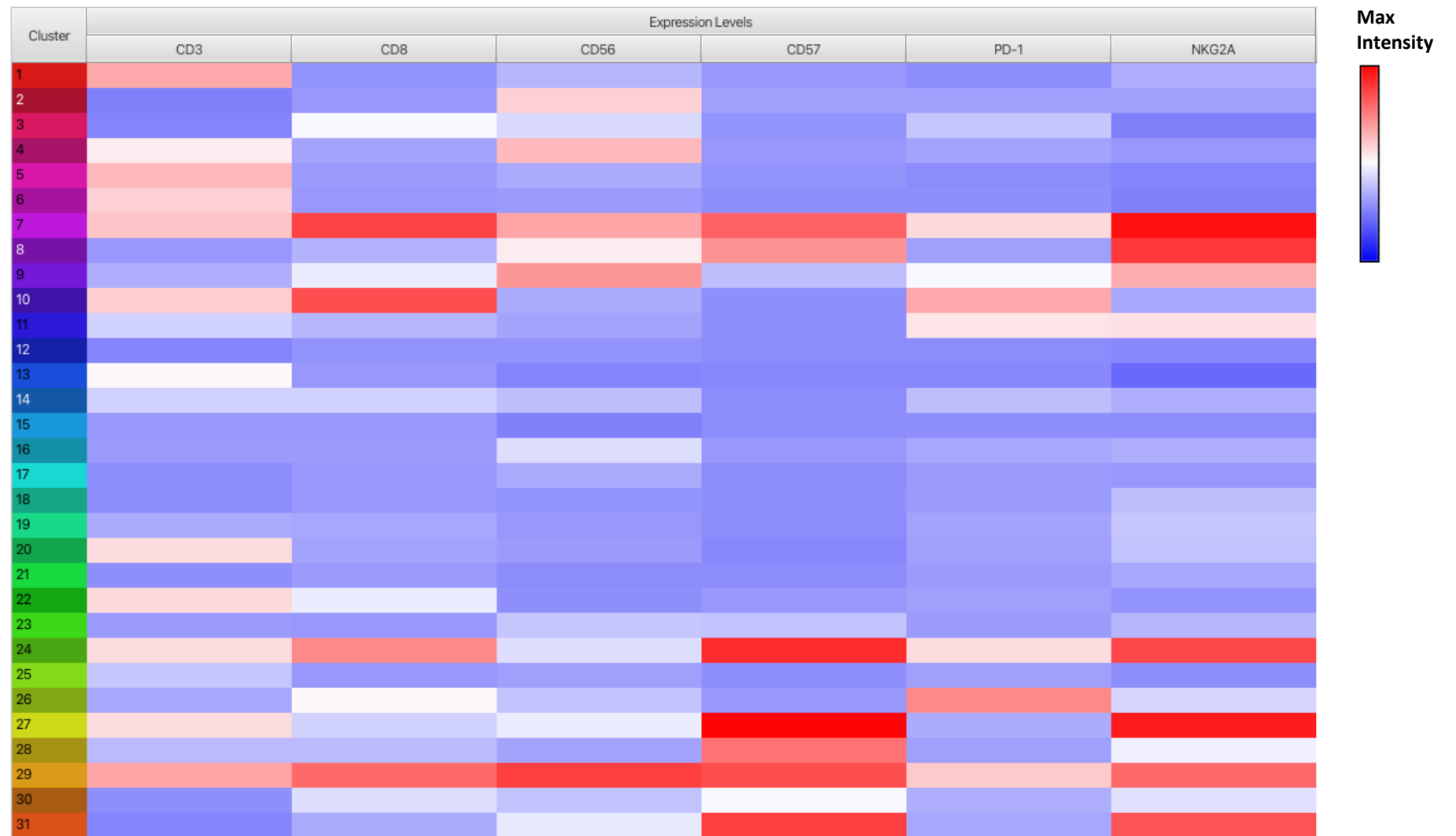

C)

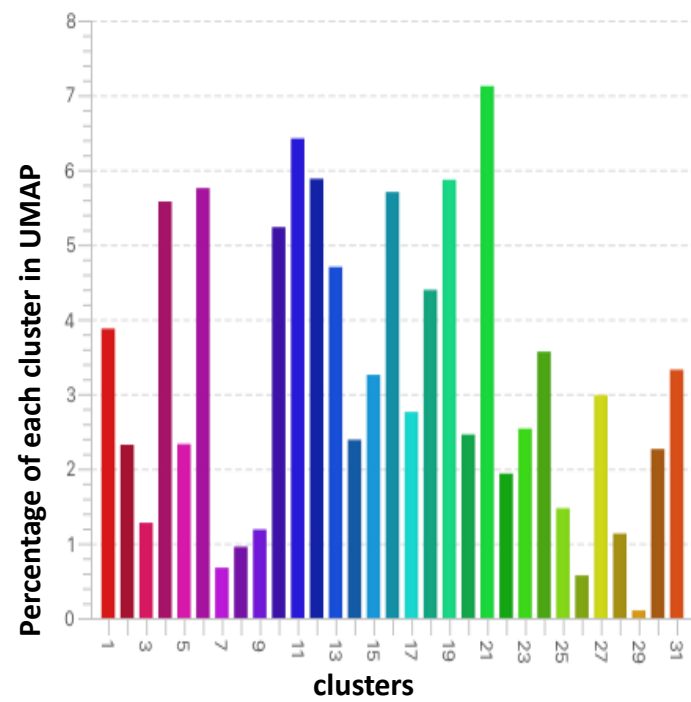

D)

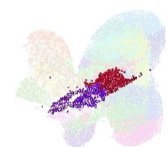

NK

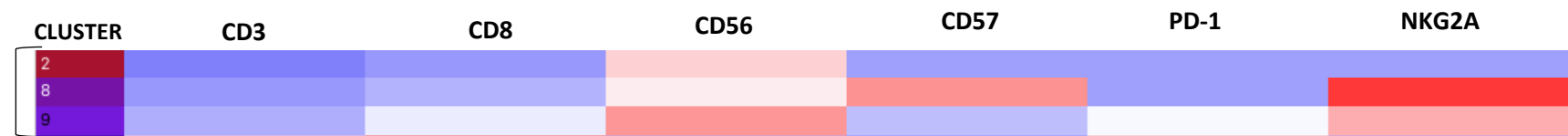

Max  
Intensity

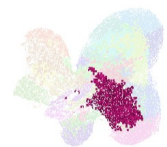

NKT

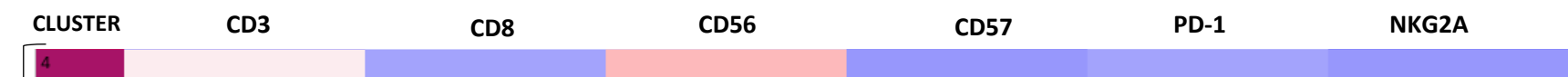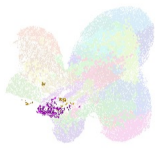

CD8/CD56

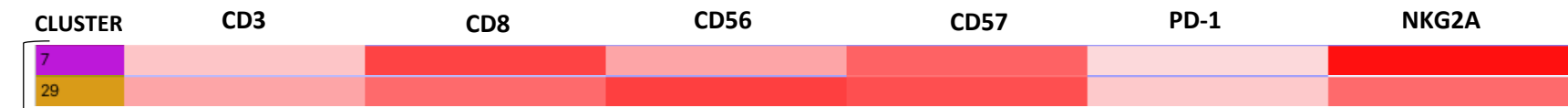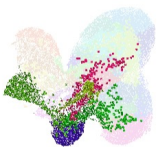

CD8

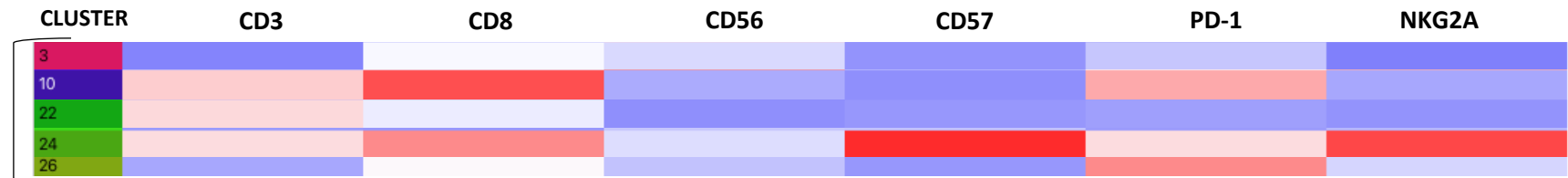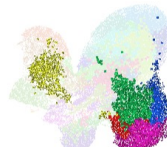

CD3+  
(high)

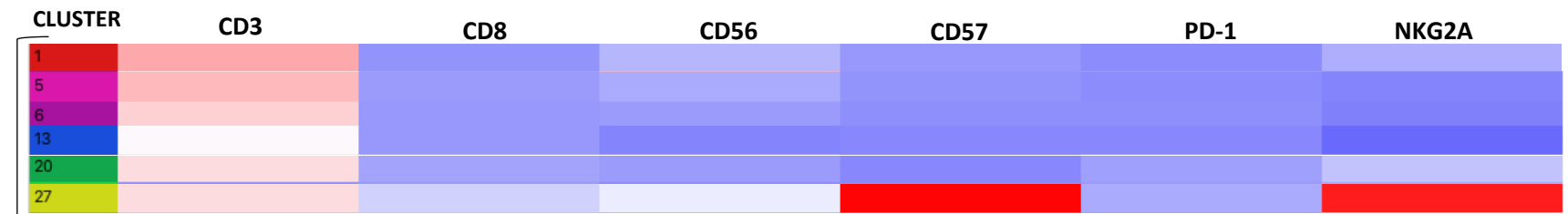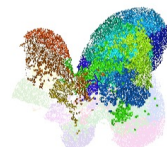

CD3-  
(low)

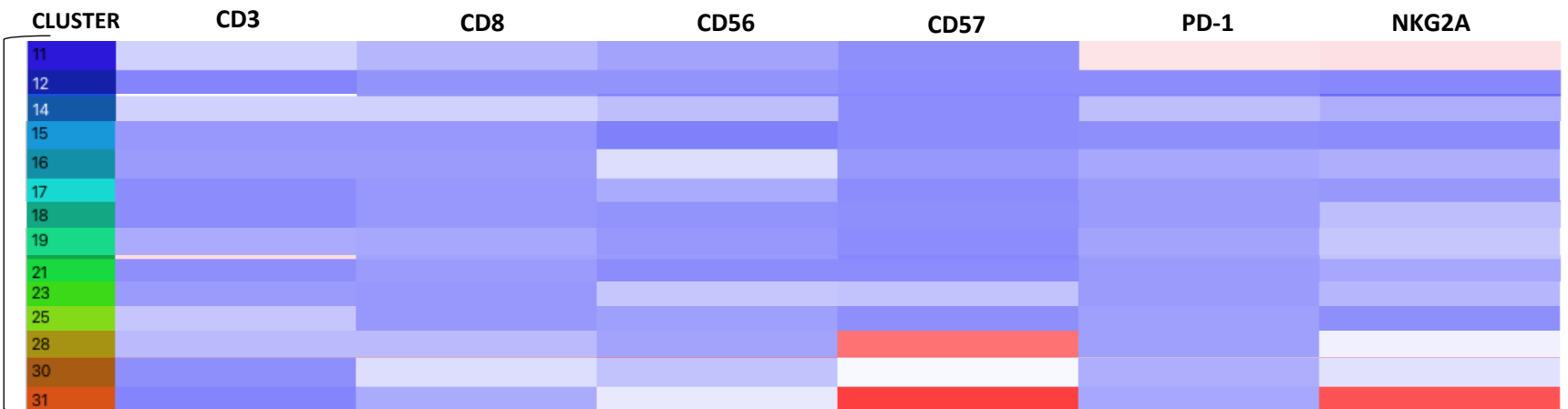

E)

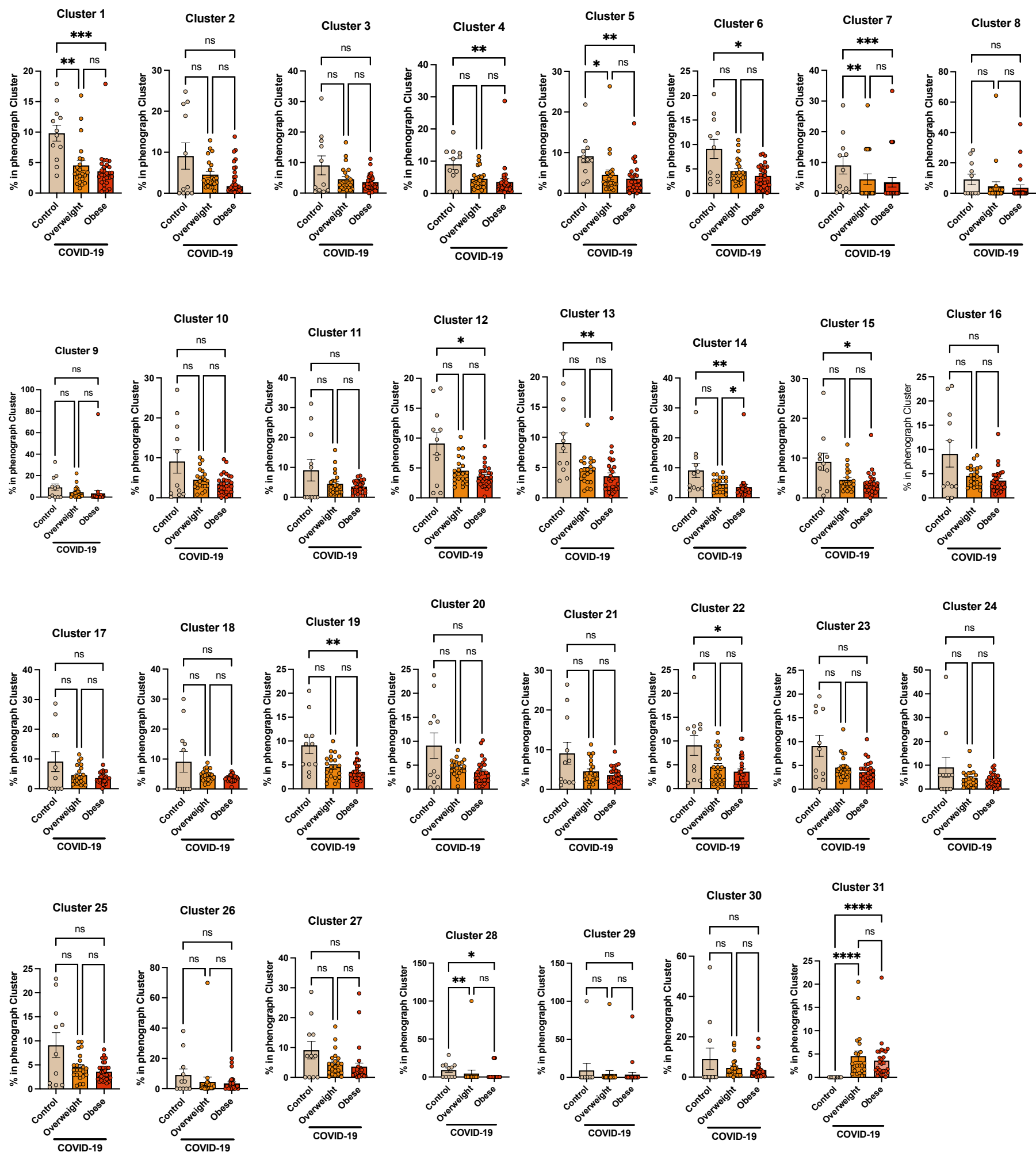

Supplement: Supplementary file 5 — Supplementary Material 5: Figure S4. Unbiased immune profiling of senescent panel. (A) The Uniform Manifold Approximation and Projection (UMAP) analysis led us to identify 31 meta clusters based on the expression of CD3, CD8, CD56, CD57, PD-1, and NKG2A molecules. (B) Heatmap displaying the relative expression (representativeness) of 31 clusters. Shades of red represent increased expression while shades of blue denote decreased expression for each indicated marker. (C) The proportion of each cluster in UMAP. (D) The 31 clusters were separated according to the indicated subsets. (E) The percentage of each cluster in Phenograph-identified clusters among the studied groups (control, overweight, and obese). Statistically significant differences are indicated: * p < 0.05, ** p < 0.01 and **** p < 0.0001 (Kruskal-Wallis, Dunn’s multiple comparison test) [file 12979_2024_423_MOESM5_ESM.pdf]
